# Manure Preferences and Postemergence Learning of Two Filth Fly Parasitoids, *Spalangia cameroni* and *Muscidifurax raptor* (Hymenoptera: Pteromalidae)

**DOI:** 10.1371/journal.pone.0167893

**Published:** 2016-12-09

**Authors:** Caitlin E. Taylor, Erika T. Machtinger, Christopher J. Geden, Matthew Kramer

**Affiliations:** 1 Animal Sciences Department, University of Florida, Gainesville, FL United States of America; 2 Entomology and Nematology Department, University of Florida, Gainesville, FL United States of America; 3 Center for Medical, Agricultural, and Veterinary Entomology, USDA-ARS, Gainesville, FL United States of America; 4 Statistics Group, USDA-ARS, Beltsville, MD United States of America; Natural Resources Canada, CANADA

## Abstract

The efficiency of host-seeking behavior is crucial to the reproductive performance of female parasitoids. Initially, parasitoids may use chemical information garnered from the microhabitat in which they emerge to locate hosts. *Spalangia cameroni* and *Muscidifurax raptor* are commercially available parasitoids of filth flies. Postemergence exposure to a specific manure may provide a way to increase parasitism in specific microhabitats found at livestock facilities upon release. In this study, female parasitoids of both species were exposed to equine manure, bovine manure, or clean pupae. Females from each emergence exposure were tested in a two-choice arena (house fly hosts in bovine manure versus clean pupae, equine manure versus clean pupae, and equine manure versus bovine manure) for progeny production. There was a detectable but small effect of postemergence exposure on *S*. *cameroni*, but it was not sufficient to reverse innate preferences. Females consistently produced more progeny in hosts found in any manure over clean pupae, and in equine manure over bovine manure. The effect of postemergence exposure on *M*. *raptor* was also detectable but small. Females produced equal numbers of progeny in bovine manure versus clean pupae, as opposed to preferring to oviposit in clean pupae as with all other treatments. Preferences by *M*. *raptor* were overall less marked than for *S*. *cameroni;* indeed most of the variability observed for this species did not result from the treatment design. Residual host mortality was also detectably altered by exposure in both species, but the effect was small. Thus, postemergence exposure did not consistently and effectively manipulate these parasitoids to producing progeny in different exposure manures, suggesting that microhabitat preferences are largely determined by other factors.

## Introduction

*Spalangia cameroni* Perkins and *Muscidifurax raptor* Girault and Sanders (Hymenoptera: Pteromalidae) are commercially available filth fly ectoparasitoids. These pupal parasitoids are used to control house flies (*Musca domestica* L.) and stable flies (*Stomoxys calcitrans* (L.)). These filth flies are of medical and veterinary importance [1], and can aggregate in large numbers becoming a challenge for livestock operations. Augmentative biological control is used for filth fly control on livestock, poultry, and equine facilities. However, past evaluation studies with various parasitoid species have documented control success in some situations, and not in others [2–9]. The variability in fly control may be a result of the limited information on the basic biology of host-seeking behavior and preferences of these parasitoids.

Parasitoid foraging is a sequential series of host-seeking behaviors including habitat location, host location, and host acceptance [10]. A parasitoid that can learn cues associated with the host microhabitat can increase its likelihood of future host location, and thereby increase reproductive success [11–12]. Newly emerged filth fly parasitoids face a challenge locating filth fly development habitats that are often ephemeral and patchily distributed. Pteromalid parasitoid responses to chemical stimuli vary with species. Some parasitoid species are attracted to host habitat [13], but are more attracted to a combination of hosts and habitat [14–17]. Other species seem to use cues directly from the hosts, with host habitat alone being somewhat repellent [18–19].

Both innate and learned cues associated with hosts and their habitat can be used by parasitoids during foraging [11]. Foraging experience may alter innate preferences, adaptively optimizing foraging efficiency [20]. Parasitoid wasps may use information acquired immediately upon emergence to locate hosts which can originate from the microhabitat in which they emerge [21–25]. This postemergence experience to host habitat can affect the host-seeking behavior of some parasitoids by modifying microhabitat preferences to those with characteristics initially experienced. Indeed, some parasitoids can be manipulated to increase or change responsiveness to specific habitats through postemergence experience [25–29]. If postemergence experience to microhabitats associated with specific livestock or other animals enhance or change preferences of pupal parasitoids of filth flies, parasitoids could be manipulated to improve their performance as biological control agents in different situations.

Olfactometer studies have shown that *Spalangia* spp. and *Muscidifurax* spp. differ in attraction to both host life stage [17], and manure type [19]. It is unknown whether postemergence exposure may change these innate odor preferences. Because *S*. *cameroni* and *M*. *raptor* often are released together, comparative studies on the effect of postemergence experience on these two extrinsic parasitoids may be used to improve their effectiveness in augmentative biological control programs [30–31]. The purpose of this study was to assess the effect of postemergence exposure of *S*. *cameroni* and *M*. *raptor* to bovine and equine manure on subsequent host-seeking and progeny production.

## Materials and Methods

### House Flies and Parasitoids

House flies came from a long-established insecticide-susceptible colony (“Orlando Normal”), originally collected in the 1950’s near Orlando, Florida. House fly hosts were kept at the USDA-ARS, Center for Medical, Agricultural and Veterinary Entomology in Gainesville, FL. Immature flies were reared on a diet consisting of 6.5 L wheat bran, 500 cc Calf Manna (Manna Pro LLC, Chesterfield, MO), and 3.8 L water. After the 6-d larval development period, puparia were collected from the medium by floating in tap water.

Two species of parasitoids were tested, *S*. *cameroni* and *M*. *raptor*, from colonies established in 2012 from wild-caught individuals on a dairy in Gilchrist County, FL. Parasitoid colonies were maintained by providing 2-d-old house fly puparia to parasitoids on a biweekly basis at a host: parasitoid ratio of at least 5:1. Both parasitoid colonies were housed in 17.5 x 17.5 x 17.5 cm BugDorm ^TM^ (MegaView Science, Taiwan) cages at 25°C and 80% RH under constant light.

### Experimental Substrates

Equine manure, collected from a private facility in High Springs, FL, and bovine manure, collected from a dairy in Bell, FL, were used for comparisons as described in Machtinger et al. [17]. Briefly, equine manure from a mare and two geldings, and bovine manure from a pen containing 3-month-old bull calves, was collected. Manure collected from both horses and cattle was <24 h post-defecation. Approximately 30 individual manure pats were collected, homogenized by hand, and then frozen at -18°C for a minimum of 1 week prior to testing to kill any existing arthropods.

A standardized amount of 200 g was used for each manure treatment. Thawed manure was placed in a 265 cm^3^ (16 oz) plastic fly rearing cup measuring 6-cm-h x 7.5-cm-diam. Equine manure was hydrated to 70% by weight [17], and mixed thoroughly. Because of previously established preferences of *S*. *cameroni* for hosts developing in manure, house fly hosts were reared in each manure sample [17]. House fly eggs (100 eggs per cup) were applied to a moistened cloth and placed on the surface of the manure [17]. Fly rearing cups were covered with muslin, sealed with plastic rim lids with the center removed, and maintained at 27°C and 80% RH under constant light. House flies were reared until pupation and pupae had turned a light reddish brown (approximately 2-d-old) before use. Clean 2-d-old pupae from the house fly colony were also placed in fly rearing cups without manure. Manure and pupae in each cup were used only once for each test.

### Substrate Bioassays

For each replicate, 5 g of parasitized house fly pupae immediately prior to expected parasitoid emergence (20-d-old pupae parasitized by *M*. *raptor* or 27-d-old pupae parasitized by *S*. *cameroni)* was placed in each of three 150 cm^3^ plastic parasitoid emergence cups measuring 6-cm-h x 7.5-cm-diam and covered with 40 g of either hydrated equine manure, bovine manure, or left uncovered (clean). Parasitoid emergence cups were covered with muslin and held at 23°C and 50% RH under constant light for approximately 1 week for parasitoid emergence.

Arenas used for host-seeking were 47.5 x 47.5 x 93-cm, and composed of 2 side panels of polyester netting (72 x 26 mesh) for ventilation and 2 sides with transparent plastic sheeting (MegaView Science, Taiwan). The contents of the fly rearing cups (clean pupae or equine or bovine manure with associated fly pupae) were emptied individually on to 21.5 cm diam paper plates (Dixie Consumer Products, LLC. Atlanta, GA), and gently spread over the surface of the plate. Two plates (treatments) were placed at either end of each arena. Trial combinations in the release arenas were composed of the following treatments: (1) pupae in equine manure vs. clean pupae, (2) pupae in bovine manure vs. clean pupae, (3) pupae in equine manure vs. pupae in bovine manure, and (4) clean pupae vs. clean pupae as a control, as well as to determine if manure exposure influences parasitism of clean pupae. In addition, subsamples of clean pupae were held in plastic cups 120 ml (4 oz) cups (Plastic Container City, Brooklyn, NY) outside the trial arenas and not exposed to parasitoids as an additional control. Placement of each treatment within each arena was done randomly.

Female parasitoids were collected from parasitoid emergence cups at 2 d (*S*. *cameroni*) or 3 days (*M*. *raptor*)after emergence by briefly cooling [17]. For each replicate, 25 *S*. *cameroni* or 20 *M*. *raptor* females from the equine manure, bovine manure, or clean pupae parasitoid emergence cup were used. Parasitoids were released in the center of each arena and given 48 hrs for host-seeking and parasitism. Pupae were recovered from the manure on each plate by floatation [32–33], and subsequently dried before placing pupae in 30 ml (1 oz) cups (Plastic Container City, Brooklyn, NY). Recovered pupae were held at 25°C and 80% RH. Adult flies were removed after emergence, and pupae were held for an additional 6 wks for parasitoid emergence. Parasitism was calculated by counting emergence holes for each species.

Each set of pupal and substrate comparisons was replicated five times for each parasitoid emergence exposure (parasitoids emerging in equine manure, bovine manure, or clean pupae) for each species.

### Statistical Analysis

Data on the number of successfully parasitized pupae were modeled separately for *S*. *cameroni* and *M*. *raptor*, and for each exposure condition-arena combination, in the generalized linear models framework as over-dispersed binomial variables using the glm function in R [34]. Data is presented in S1 Table. Most biological data generated by binomial-like processes are over-dispersed, typically because individual counts are not independent. In this case, over-dispersion was likely due to parasitoids first making a substrate choice and then searching for pupae in that substrate in which to oviposit. For this analysis the dependent variable was the count of parasitoid progeny for each of the two choices. The independent variables were the exposure treatments (equine manure, bovine manure, no manure) and the paired pupal presentations (arena). A similar analysis was conducted on the number of pupae which produced neither fly nor parasitoid (residual host mortality). Residual host mortality is an important consideration in biological control [35] and may occur due to destructive host feeding [36], ovipositor probing and/or chemical injection [37–40], or failed development of parasitoid offspring [41]. An additional analysis was performed that included species (and its interaction with other effects), to better understand how species differ; here the independent variable was the paired counts of successfully parasitized pupae versus other outcomes. Finally, we performed a standard variance decomposition for linear models, using arcsine-square root transformed ratios of the count of parasitoid progeny of one choice (e.g., equine manure) to the sum of total parasitoid progeny in the arena as the dependent variable, to estimate how much each of the independent variables (and their interactions) influenced choice, using the lme4 package [42] in R, where we considered all independent variables as random effects. Experiment-wise error (over the whole study) was not controlled, i.e. significance was declared at α = 0.05, throughout, though most *p*-values were much smaller, as is appropriate in exploratory studies where type II error control is more important.

## Results

**Fig 1:**
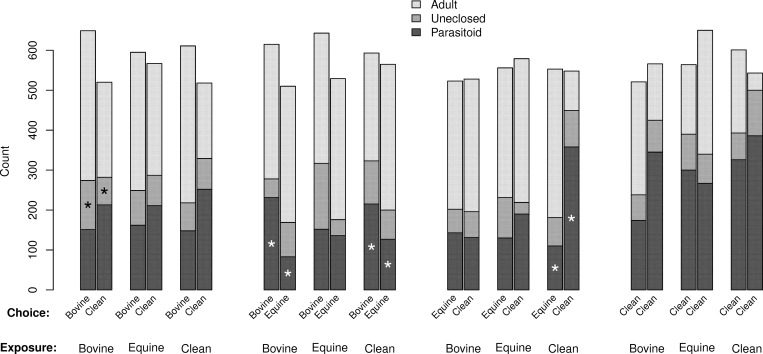
Frequencies of outcomes of house fly host pupa in paired choice tests for 100 female *Muscidifurax raptor* parasitoids (five replications of 20). Paired bars give choices, with shading giving the three possible outcomes (adult = adult fly emerges, uneclosed = nothing emerges, parasitoid = parasitoid emerges). Parasitoids were exposed to one of three conditions prior to testing (bovine manure, equine manure, no manure = clean). Asterisks in paired bars indicate significant differences in counts (choice tested as a 1-parameter contrast in a generalized linear model assuming an over-dispersed binomial distribution).

**Fig 2:**
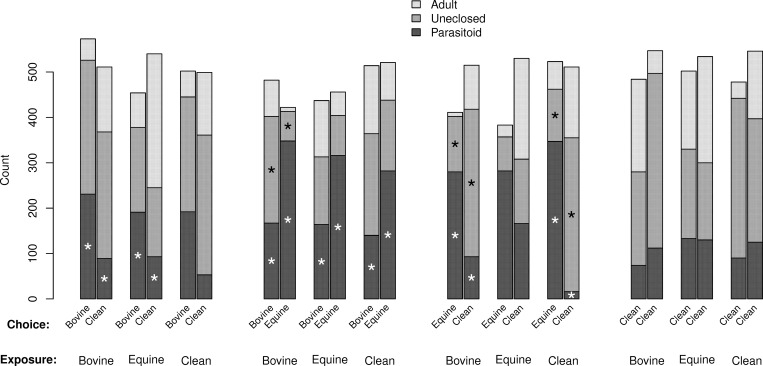
Frequencies of outcomes of house fly host pupa in paired choice tests for 125 female *Spalangia cameroni* parasitoids (five replications of 25). Paired bars give choices, with shading giving the three possible outcomes (adult = adult fly emerges, uneclosed = nothing emerges, parasitoid = parasitoid emerges). Parasitoids were exposed to one of three conditions prior to testing (bovine manure, equine manure, no manure = clean). Asterisks in paired bars indicate significant differences in counts (choice tested as a 1-parameter contrast in a generalized linear model assuming an over-dispersed binomial distribution).

No parasitoids emerged from control pupae held outside the release arenas. Counts and results of statistical tests (probabilities for parameter estimates based on *t*-statistics) for *M*. *raptor* are given in Fig 1, those for *S*. *cameroni* are given in Fig 2. There was marked evidence of over-dispersion in most comparisons (ranging from 1.0 [no over-dispersion] to 31.1 [substantial over-dispersion], most were greater than three). We interpret the over-dispersion to represent the non-independence of pupa selected for egg deposition by individual wasps; females likely chose pupa close by (on the same side of the arena) when sequentially laying eggs.

There was some influence of initial exposure for *M*. *raptor*, that is, which side of the arena was chosen for oviposition was affected by prior exposure for some combinations of exposure and choice (Fig 1). There were significantly more parasitoid progeny in the bovine manure vs. equine manure if the initial exposure was bovine manure (*t*-test, 4 d.f., *p* = 0.0210) or clean (*t*-test, 4 d.f., *p* = 0.0007). There were also significantly more parasitoid progeny in the clean choice (versus equine) if the initial exposure was clean (*t*-test, 4 d.f., *p* = 0.0105). Counts and results of statistical tests for *Spalangia cameroni* are given in Fig 2. This species showed overall stronger differences in progeny number, especially if one choice was equine manure. The only evidence for an influence of exposure was the bovine-clean choice, where those exposed to clean did not show a significance preference for bovine (*t*-test, 4 d.f., *p* = 0.0590; note that the over-dispersion parameter was estimated to be large, 10.04, for this test), and their preference for equine over clean was not significant if the initial exposure was to equine manure (*t*-test, 4 d.f., *p* = 0.3139; note that this contradicts expectations that exposure to a substrate increases preference for that substrate). For the uneclosed (residual mortality) dependent variable, there were fewer significant differences in counts, and for these, three appear to be the inverse of the successful parasitoid progeny variable (i.e. more successful parasitoid progeny were associated with fewer uneclosed pupa).

To examine species differences, we looked at results from a statistical model that included species and looked at interaction terms that included species. Three were significant; initial exposure (χ^2^ test, 2 d.f., *p* = 0.0018), arena (kind of choice, χ^2^ test, 3 d.f., *p* < 0.0001), and choice (χ^2^ test, 2 d.f., *p* < 0.0001). Thus, pupal seeking and/or egg deposition behavior in the two species differed in several dimensions, most notably in the stronger and apparently less modifiable preferences exhibited by *S*. *cameroni*. It also appears to be the parasitoid that better prevents adult flies from emerging, even taking into account the slightly larger number of *S*. *cameroni* released into the arena; note that the number of emerging adult flies is considerably higher for *M*. *raptor* in most tests except those where only clean pupa were offered (compare Figs 1 and 2 for 'Adult').

The variance decompositions (Table 1) again demonstrate the species differences for several variance components. For *M*. *raptor*, the sum of all treatment effects (i.e. everything other than the residual) is 18% of the total variance. Of the treatment effects, only choice (wasps did not choose sides randomly) and the exposure by arena (type of choice offered) interaction (the exposure effect differed between arenas) were important. The residual is the sum of binomial sampling error (relatively small given the large number of counts) and over-dispersion. Over-dispersion was somewhat larger for *M*. *raptor* than for *S*. *cameroni* (compare the residual estimate of 0.0603 with 0.0394). This over-dispersion should be interpreted as non-independence of the count of parasitoid progeny from each side of the arena from an individual female parasitoid due to her searching and/or laying behavior (see Discussion).

**Table 1:** Variance decomposition of the arcsine-square root transformed ratio of parasitoid progeny for one of the two choices (e.g. equine manure) offered to all parasitoid progeny in the arena for each tested parasitoid species, *Muscidifurax raptor* and *Spalangia cameroni*.

Source of Variation^1^	*M*. *raptor*		*S*. *cameroni*	
	Variance Component	Percent	Variance Component	Percent
Exposure	0.00000	0.0	0.00000	0.0
Arena type	0.00006	0.1	0.01840	17.1
Choice	0.00840	10.4	0.00000	0.0
Arena x Choice	0.00153	1.3	0.04091	38.1
Exposure x Choice	0.00000	0.0	0.00868	8.1
Exposure x Arena	0.00495	6.1	0.00000	0.0
Unexplained (Residual)	0.06603	82.0	0.03936	36.7

^1^"Choice" is the side selected by the wasps (each arena has two choices), "Arena" is one of three types of choice offered, and "Unexplained" or "Residual" is the sum of binomial sampling error and over-dispersion.

For *S*. *cameroni*, treatment effects were stronger, indicated by a residual variance of 37% (compared to 82% for *M*. *raptor*). While the number of progeny differed depending on the arena (which two choices were available; arena was 17% percent of the total variance), the largest source of variation was the choice by arena interaction (38%). That is, the differences in the number of progeny between the two sides depended on which choices were offered. For *S*. *cameroni*, exposure also affected how large the differences were between the two choices. The exposure by arena interaction variance component was estimated to be zero.

## Discussion

Early adult experience (postemergence learning) plays a role in determining the behavior of many parasitoid species [43]. Given that both *M*. *raptor* and *S*. *cameroni* are generalist parasitoids [44–45], females must sort through a variety of stimuli to locate hosts. Parasitoids that learn to recognize cues associated with host microhabitat should be able to increase reproductive success by increasing the likelihood of future host location. The results of this study suggest some species-level differences on postemergence learning on subsequent parasitism and residual host mortality. However, these were not large effects. Postemergence learning did not substantially influence host-seeking, as measured by progeny production, for either *M*. *raptor* or *S*. *cameroni* in different manure microhabitats. Nevertheless, parasitoid progeny numbers were somewhat affected by exposure, suggesting that the influence of some postemergence experiences on host-seeking benefits both species.

The generally large over-dispersion parameters estimated from the statistical models suggest that individual parasitoids tend to select locally clustered hosts, resulting in most or all eggs being deposited in whichever side is first chosen. One prediction of the Marginal Value Theorem [46] suggests that female parasitoids will spend more time at a patch that is higher quality. Mechanistic assumptions for the process of remaining or leaving a patch have been proposed based on initial attraction in the form of kairomones, and progressive habituation to the stimulus [47–48]. Perhaps parasitoids in the current study, after initial attraction to the preferred habitat odor, encountered a suitable host and deposited an egg. After that successful oviposition experience, and the availability of more hosts than the wasps could parasitize, there would be no pressure for wasps to investigate both plates of provided hosts. This would have lead to non-independence between eggs laid by the same parasitoid, creating a clustering effect. Many factors could influence mechanistic patch-leaving rules in this case including genetic variability [42], host distance within a patch [49], between-patch travel time [50–51], kairomones [52–53], host density and distribution [54–55], and the presence of conspecific females [56]. Equipment that tracks insect movement in confined areas might be used to better understand how these parasitoids make their initial choice, and subsequent host-seeking behavior under a variety of conditions.

*Spalangia cameroni* produced more progeny in hosts found in both animal manures over clean hosts regardless of emergence experience. This species also produced greater numbers of progeny in hosts located in equine manure over all other alternatives. These results support previous studies where *S*. *cameroni* demonstrated odor preference for equine manure over bovine manure, but odors from either manure over clean air [17]. Moreover, field recoveries of parasitoids on equine facilities have been almost exclusively *Spalangia* spp. [57–59]. Taken together, these results indicate the suitability of *S*. *cameroni* for fly control on equine farms.

Similar to other odor preference studies [17], *M*. *raptor* produced greater numbers of progeny in clean pupae regardless of equine or clean pupae exposure. Postemergence experience to bovine manure did not make bovine manure a preferred microhabitat for parasitism, but seemingly altered the dominant preference for producing progeny in clean pupae. After bovine exposure, the number of progeny produced from hosts in bovine and equine manure was not different from those produced in the clean pupae alternative. *Muscidifurax raptor* superficially forages for hosts [7, 60]. Postemergence experience may have changed the behavior of this species by increasing the amount of time this parasitoid spent foraging in this complex manure environment, thus increasing the likelihood of host encounters and greater parasitism [61]. Because *M*. *raptor* appears to require a latent period postemergence before responding to odor tests [17], longer postemergence exposure may have increased initial host-finding and microhabitat acceptance. Given the large over-dispersion parameters, perhaps our results can be best explained by parasitoids in the arena making an initial choice based on airborne chemical cues and subsequently depositing most eggs in the chosen area, rather than fully investigating the entire arena prior to depositing each egg. This may be the reason these results are in agreement with earlier odor preference studies.

Current literature suggests the absence of postemergence learning is an infrequent anomaly among parasitoids, but has been observed in other species. Some *Cotesia* spp. (Hymenoptera: Braconidae) appear to lack the ability for early adult conditioning and rely on genetically fixed preferences [62–63]. The responses of *S*. *cameroni* and *M*. *raptor* appear to have large innate components. Although *M*. *raptor* and *S*. *cameroni* have a wide host range, they are often competing for the same hosts and innate responses towards specific microhabitat types and different foraging strategies may be an adaptation to mitigate the effects of resource competition. Past studies have suggested that *M*. *raptor* and *S*. *cameroni* may reconcile niche competition by seeking hosts at different ages, and by having different microhabitat preferences [17, 19]. *Spalangia cameroni* will seek hosts in less preferred microhabitat at high parasitoid densities [32], but when not pressured, this species may spend more time thoroughly foraging in specific microhabitats than *M*. *raptor* as a mechanism to avoid competition. Therefore, as observed in the current study, these innate preferences may not be easily manipulated. Short-lived parasitoids in ephemeral habitats also may have fitness penalties when learning is relied upon if the microhabitats experienced at emergence are not available. Learning could be less efficient than reliable, innate information. Previous research has demonstrated the attraction of *S*. *cameroni* to developing filth fly larvae over pupae [17] and preference induction may require the presence of host odors along with manure, not provided in our pre-trial exposure. It remains to be seen what specific cues are used by these parasitoids in host-seeking that could further elucidate behavior and host-seeking preferences.

Residual host mortality differed based on postemergence exposure. More residual host mortality was induced by *M*. *raptor* in hosts in the manure treatment adult wasps were exposed to. This may suggest that exposure may influence host location and foraging, but not progeny production. Compounds from hosts, such as hydrocarbons, can elicit reflexive ovipositor piercing or probing [64–65].

Female parasitoids might locate hosts and forage longer in the exposure microhabitat, and exhibit probing behavior that may kill hosts, but may still prefer ovipositing in clean pupae and not consider hosts in manure suitable for offspring. Alternatively, compounds from manure may inhibit oviposition. Interestingly, more progeny were produced in clean pupae in the control arenas that did not contain manure than nearly all other test arenas.

*Spalangia cameroni* only induced greater residual host mortality in hosts in bovine manure over equine manure when exposed to bovine manure, and in clean pupae when compared against hosts in equine manure. It is likely that in equine manure comparisons, because equine manure appears to be an innately preferred microhabitat, the difference in residual host mortality was a result of more progeny being produced in hosts in equine manure in lieu of other mortality causes, which may infer that equine manure is assessed by the *S*. *cameroni* as being a high quality habitat. Residual host mortality and parasitism may have been affected by mutual interference of competing parasitoids, though not all pupae were parasitized in each treatment. From a biological control perspective, residual host mortality is important and as desirable as progeny production in innundative releases [35]. Because there was more observed residual mortality produced by *S*. *cameroni* than *M*. *raptor*, this particular results should be further explored to assess the overall benefit of these two species in biological control programs.

Both *S*. *cameroni* and *M*. *raptor* are valuable biological control agents of filth flies. A better understanding of the cues used by these parasitoids to locate and kill hosts could improve the use of these parasitoids to suppress fly populations. In theory, preference induction by exposing parasitoids to different microhabitats at adult emergence could be useful to increase parasitism in targeted fly development areas. However, even after postemergence exposure to different animal manures, these parasitoids did not overwhelmingly produce progeny in the manure to which they were exposed, suggesting that innate microhabitat preferences generally trump flexibility acquired through learning. The observed behavior of these parasitoids, both in microhabitat preference and host location preference, instead support the release of multiple species for fly control.

## Supporting Information

S1 Table Raw data results of parasitoid progeny, residual host mortality, and emerged adult flies. (DOCX)
