# Supplementary material for: Manure Preferences and Postemergence Learning of Two Filth Fly Parasitoids, Spalangia cameroni and Muscidifurax raptor (Hymenoptera: Pteromalidae)
Source: PLoS One. 2016 Dec 9;11(12):e0167893. doi: 10.1371/journal.pone.0167893 (PMC5148001; doi:10.1371/journal.pone.0167893)
Supplement: S1 Table — (DOCX) [file pone.0167893.s001.docx]

| RaParasitoid Treatment | Species | Treatment Number | Arena | Side | Adult | Uneclosed | Parasitoid | Total Pupae | Total Uneclosed pupae in treatment in plate | Total Uneclosed pupae in cage |
| --- | --- | --- | --- | --- | --- | --- | --- | --- | --- | --- |
| Bovine | Mr | 1 | Bovine V. Naked | Bovine | 31 | 29 | 66 | 126 | 95 | 275 |
| Bovine | Mr | 2 | Bovine V. Naked | Bovine | 110 | 28 | 11 | 149 | 39 | 275 |
| Bovine | Mr | 3 | Bovine V. Naked | Bovine | 82 | 11 | 22 | 115 | 33 | 259 |
| Bovine | Mr | 4 | Bovine V. Naked | Bovine | 95 | 26 | 23 | 144 | 49 | 259 |
| Bovine | Mr | 5 | Bovine V. Naked | Bovine | 57 | 29 | 29 | 115 | 58 | 218 |
| Bovine | Mr | 1 | Bovine V. Naked | Naked | 61 | 16 | 26 | 103 | 42 | 218 |
| Bovine | Mr | 2 | Bovine V. Naked | Naked | 39 | 22 | 44 | 105 | 66 | 208 |
| Bovine | Mr | 3 | Bovine V. Naked | Naked | 93 | 7 | 3 | 103 | 10 | 208 |
| Bovine | Mr | 4 | Bovine V. Naked | Naked | 41 | 14 | 49 | 104 | 63 | 209 |
| Bovine | Mr | 5 | Bovine V. Naked | Naked | 4 | 10 | 91 | 105 | 101 | 209 |
| Bovine | Mr | 1 | Equine V. Bovine | Bovine | 75 | 17 | 52 | 144 | 69 | 262 |
| Bovine | Mr | 2 | Equine V. Bovine | Bovine | 69 | 2 | 47 | 118 | 49 | 262 |
| Bovine | Mr | 3 | Equine V. Bovine | Bovine | 69 | 9 | 37 | 115 | 46 | 259 |
| Bovine | Mr | 4 | Equine V. Bovine | Bovine | 81 | 7 | 56 | 144 | 63 | 259 |
| Bovine | Mr | 5 | Equine V. Bovine | Bovine | 43 | 12 | 39 | 94 | 51 | 194 |
| Bovine | Mr | 1 | Equine V. Bovine | Equine | 65 | 11 | 24 | 100 | 35 | 194 |
| Bovine | Mr | 2 | Equine V. Bovine | Equine | 61 | 15 | 26 | 102 | 41 | 229 |
| Bovine | Mr | 3 | Equine V. Bovine | Equine | 74 | 34 | 19 | 127 | 53 | 229 |
| Bovine | Mr | 4 | Equine V. Bovine | Equine | 89 | 11 | 4 | 104 | 15 | 181 |
| Bovine | Mr | 5 | Equine V. Bovine | Equine | 52 | 15 | 10 | 77 | 25 | 181 |
| Bovine | Mr | 1 | Equine V. Naked | Equine | 38 | 15 | 54 | 107 | 69 | 211 |
| Bovine | Mr | 2 | Equine V. Naked | Equine | 81 | 5 | 18 | 104 | 23 | 211 |
| Bovine | Mr | 3 | Equine V. Naked | Equine | 65 | 9 | 34 | 108 | 43 | 220 |
| Bovine | Mr | 4 | Equine V. Naked | Equine | 79 | 18 | 15 | 112 | 33 | 220 |
| Bovine | Mr | 5 | Equine V. Naked | Equine | 58 | 12 | 22 | 92 | 34 | 199 |
| Bovine | Mr | 1 | Equine V. Naked | Naked | 64 | 17 | 26 | 107 | 43 | 199 |
| Bovine | Mr | 2 | Equine V. Naked | Naked | 84 | 6 | 15 | 105 | 21 | 208 |
| Bovine | Mr | 3 | Equine V. Naked | Naked | 95 | 4 | 4 | 103 | 8 | 208 |
| Bovine | Mr | 4 | Equine V. Naked | Naked | 83 | 12 | 17 | 112 | 29 | 213 |
| Bovine | Mr | 5 | Equine V. Naked | Naked | 6 | 26 | 69 | 101 | 95 | 213 |
| Bovine | Mr | 1 | Naked V. Naked | Control | 1 | 16 | 90 | 107 | 106 | 211 |
| Bovine | Mr | 1 | Naked V. Naked | Control | 42 | 19 | 43 | 104 | 62 | 211 |
| Bovine | Mr | 2 | Naked V. Naked | Control | 71 | 12 | 21 | 104 | 33 | 206 |
| Bovine | Mr | 2 | Naked V. Naked | Control | 72 | 10 | 20 | 102 | 30 | 206 |
| Bovine | Mr | 3 | Naked V. Naked | Control | 97 | 7 | 0 | 104 | 7 | 210 |
| Bovine | Mr | 3 | Naked V. Naked | Control | 47 | 16 | 43 | 106 | 59 | 210 |
| Bovine | Mr | 4 | Naked V. Naked | Control | 21 | 20 | 69 | 110 | 89 | 239 |
| Bovine | Mr | 4 | Naked V. Naked | Control | 55 | 5 | 69 | 129 | 74 | 239 |
| Bovine | Mr | 5 | Naked V. Naked | Control | 12 | 16 | 86 | 114 | 102 | 221 |
| Bovine | Mr | 5 | Naked V. Naked | Control | 6 | 23 | 78 | 107 | 101 | 221 |
| Equine | Mr | 1 | Bovine V. Naked | Bovine | 77 | 4 | 32 | 113 | 36 | 221 |
| Equine | Mr | 2 | Bovine V. Naked | Bovine | 82 | 1 | 25 | 108 | 26 | 221 |
| Equine | Mr | 3 | Bovine V. Naked | Bovine | 49 | 50 | 37 | 136 | 87 | 274 |
| Equine | Mr | 4 | Bovine V. Naked | Bovine | 76 | 18 | 44 | 138 | 62 | 274 |
| Equine | Mr | 5 | Bovine V. Naked | Bovine | 62 | 14 | 24 | 100 | 38 | 212 |
| Equine | Mr | 1 | Bovine V. Naked | Naked | 56 | 18 | 38 | 112 | 56 | 212 |
| Equine | Mr | 2 | Bovine V. Naked | Naked | 100 | 8 | 0 | 108 | 8 | 215 |
| Equine | Mr | 3 | Bovine V. Naked | Naked | 8 | 20 | 79 | 107 | 99 | 215 |
| Equine | Mr | 4 | Bovine V. Naked | Naked | 77 | 13 | 41 | 131 | 54 | 240 |
| Equine | Mr | 5 | Bovine V. Naked | Naked | 39 | 17 | 53 | 109 | 70 | 240 |
| Equine | Mr | 1 | Equine V. Bovine | Bovine | 52 | 21 | 23 | 96 | 44 | 241 |
| Equine | Mr | 2 | Equine V. Bovine | Bovine | 90 | 23 | 32 | 145 | 55 | 241 |
| Equine | Mr | 3 | Equine V. Bovine | Bovine | 60 | 51 | 35 | 146 | 86 | 285 |
| Equine | Mr | 4 | Equine V. Bovine | Bovine | 67 | 36 | 36 | 139 | 72 | 285 |
| Equine | Mr | 5 | Equine V. Bovine | Bovine | 57 | 34 | 26 | 117 | 60 | 210 |
| Equine | Mr | 1 | Equine V. Bovine | Equine | 61 | 6 | 26 | 93 | 32 | 210 |
| Equine | Mr | 2 | Equine V. Bovine | Equine | 64 | 24 | 16 | 104 | 40 | 202 |
| Equine | Mr | 3 | Equine V. Bovine | Equine | 58 | 1 | 39 | 98 | 40 | 202 |
| Equine | Mr | 4 | Equine V. Bovine | Equine | 62 | 3 | 35 | 100 | 38 | 234 |
| Equine | Mr | 5 | Equine V. Bovine | Equine | 108 | 6 | 20 | 134 | 26 | 234 |
| Equine | Mr | 1 | Equine V. Naked | Equine | 72 | 3 | 30 | 105 | 33 | 207 |
| Equine | Mr | 2 | Equine V. Naked | Equine | 57 | 3 | 42 | 102 | 45 | 207 |
| Equine | Mr | 3 | Equine V. Naked | Equine | 60 | 24 | 21 | 105 | 45 | 224 |
| Equine | Mr | 4 | Equine V. Naked | Equine | 68 | 48 | 3 | 119 | 51 | 224 |
| Equine | Mr | 5 | Equine V. Naked | Equine | 68 | 23 | 34 | 125 | 57 | 244 |
| Equine | Mr | 1 | Equine V. Naked | Naked | 90 | 6 | 23 | 119 | 29 | 244 |
| Equine | Mr | 2 | Equine V. Naked | Naked | 52 | 7 | 51 | 110 | 58 | 220 |
| Equine | Mr | 3 | Equine V. Naked | Naked | 66 | 4 | 40 | 110 | 44 | 220 |
| Equine | Mr | 4 | Equine V. Naked | Naked | 88 | 6 | 36 | 130 | 42 | 240 |
| Equine | Mr | 5 | Equine V. Naked | Naked | 64 | 6 | 40 | 110 | 46 | 240 |
| Equine | Mr | 1 | Naked V. Naked | Control | 35 | 29 | 59 | 123 | 88 | 237 |
| Equine | Mr | 1 | Naked V. Naked | Control | 38 | 19 | 57 | 114 | 76 | 237 |
| Equine | Mr | 2 | Naked V. Naked | Control | 66 | 12 | 28 | 106 | 40 | 215 |
| Equine | Mr | 2 | Naked V. Naked | Control | 10 | 6 | 93 | 109 | 99 | 215 |
| Equine | Mr | 3 | Naked V. Naked | Control | 25 | 24 | 63 | 112 | 87 | 218 |
| Equine | Mr | 3 | Naked V. Naked | Control | 25 | 20 | 61 | 106 | 81 | 218 |
| Equine | Mr | 4 | Naked V. Naked | Control | 96 | 11 | 48 | 155 | 59 | 295 |
| Equine | Mr | 4 | Naked V. Naked | Control | 96 | 2 | 42 | 140 | 44 | 295 |
| Equine | Mr | 5 | Naked V. Naked | Control | 8 | 28 | 70 | 106 | 98 | 249 |
| Equine | Mr | 5 | Naked V. Naked | Control | 85 | 12 | 46 | 143 | 58 | 249 |
| Naked | Mr | 1 | Bovine V. Naked | Bovine | 92 | 0 | 40 | 132 | 40 | 266 |
| Naked | Mr | 2 | Bovine V. Naked | Bovine | 88 | 19 | 27 | 134 | 46 | 266 |
| Naked | Mr | 3 | Bovine V. Naked | Bovine | 87 | 5 | 26 | 118 | 31 | 246 |
| Naked | Mr | 4 | Bovine V. Naked | Bovine | 77 | 23 | 28 | 128 | 51 | 246 |
| Naked | Mr | 5 | Bovine V. Naked | Bovine | 49 | 23 | 27 | 99 | 50 | 201 |
| Naked | Mr | 1 | Bovine V. Naked | Naked | 100 | 2 | 0 | 102 | 2 | 201 |
| Naked | Mr | 2 | Bovine V. Naked | Naked | 26 | 25 | 48 | 99 | 73 | 213 |
| Naked | Mr | 3 | Bovine V. Naked | Naked | 24 | 19 | 71 | 114 | 90 | 213 |
| Naked | Mr | 4 | Bovine V. Naked | Naked | 37 | 11 | 56 | 104 | 67 | 203 |
| Naked | Mr | 5 | Bovine V. Naked | Naked | 2 | 20 | 77 | 99 | 97 | 203 |
| Naked | Mr | 1 | Equine V. Bovine | Bovine | 77 | 9 | 48 | 134 | 57 | 268 |
| Naked | Mr | 2 | Equine V. Bovine | Bovine | 55 | 29 | 50 | 134 | 79 | 268 |
| Naked | Mr | 3 | Equine V. Bovine | Bovine | 75 | 17 | 24 | 116 | 41 | 232 |
| Naked | Mr | 4 | Equine V. Bovine | Bovine | 46 | 20 | 50 | 116 | 70 | 232 |
| Naked | Mr | 5 | Equine V. Bovine | Bovine | 17 | 33 | 43 | 93 | 76 | 200 |
| Naked | Mr | 1 | Equine V. Bovine | Equine | 76 | 0 | 31 | 107 | 31 | 200 |
| Naked | Mr | 2 | Equine V. Bovine | Equine | 71 | 12 | 32 | 115 | 44 | 224 |
| Naked | Mr | 3 | Equine V. Bovine | Equine | 62 | 31 | 16 | 109 | 47 | 224 |
| Naked | Mr | 4 | Equine V. Bovine | Equine | 79 | 8 | 26 | 113 | 34 | 234 |
| Naked | Mr | 5 | Equine V. Bovine | Equine | 77 | 22 | 22 | 121 | 44 | 234 |
| Naked | Mr | 1 | Equine V. Naked | Equine | 72 | 15 | 26 | 113 | 41 | 245 |
| Naked | Mr | 2 | Equine V. Naked | Equine | 76 | 20 | 36 | 132 | 56 | 245 |
| Naked | Mr | 3 | Equine V. Naked | Equine | 67 | 13 | 19 | 99 | 32 | 212 |
| Naked | Mr | 4 | Equine V. Naked | Equine | 81 | 9 | 23 | 113 | 32 | 212 |
| Naked | Mr | 5 | Equine V. Naked | Equine | 76 | 14 | 6 | 96 | 20 | 232 |
| Naked | Mr | 1 | Equine V. Naked | Naked | 80 | 6 | 50 | 136 | 56 | 232 |
| Naked | Mr | 2 | Equine V. Naked | Naked | 7 | 23 | 73 | 103 | 96 | 208 |
| Naked | Mr | 3 | Equine V. Naked | Naked | 2 | 22 | 81 | 105 | 103 | 208 |
| Naked | Mr | 4 | Equine V. Naked | Naked | 3 | 20 | 81 | 104 | 101 | 204 |
| Naked | Mr | 5 | Equine V. Naked | Naked | 7 | 20 | 73 | 100 | 93 | 204 |
| Naked | Mr | 1 | Naked V. Naked | Control | 36 | 6 | 90 | 132 | 96 | 262 |
| Naked | Mr | 1 | Naked V. Naked | Control | 90 | 8 | 32 | 130 | 38 | 262 |
| Naked | Mr | 2 | Naked V. Naked | Control | 3 | 17 | 85 | 105 | 102 | 221 |
| Naked | Mr | 2 | Naked V. Naked | Control | 40 | 23 | 53 | 116 | 76 | 221 |
| Naked | Mr | 3 | Naked V. Naked | Control | 39 | 13 | 66 | 118 | 79 | 227 |
| Naked | Mr | 3 | Naked V. Naked | Control | 3 | 35 | 71 | 109 | 106 | 227 |
| Naked | Mr | 4 | Naked V. Naked | Control | 7 | 18 | 84 | 109 | 102 | 219 |
| Naked | Mr | 4 | Naked V. Naked | Control | 28 | 18 | 64 | 110 | 82 | 219 |
| Naked | Mr | 5 | Naked V. Naked | Control | 0 | 27 | 88 | 115 | 115 | 215 |
| Naked | Mr | 5 | Naked V. Naked | Control | 5 | 16 | 79 | 100 | 95 | 215 |
| Bovine | Sc | 1 | Bovine V. Naked | Bovine | 2 | 23 | 21 | 46 | 53 | 186 |
| Bovine | Sc | 2 | Bovine V. Naked | Bovine | 14 | 86 | 40 | 140 | 100 | 186 |
| Bovine | Sc | 3 | Bovine V. Naked | Bovine | 18 | 21 | 25 | 64 | 49 | 207 |
| Bovine | Sc | 4 | Bovine V. Naked | Bovine | 5 | 76 | 62 | 143 | 113 | 207 |
| Bovine | Sc | 5 | Bovine V. Naked | Bovine | 8 | 89 | 83 | 180 | 124 | 285 |
| Bovine | Sc | 1 | Bovine V. Naked | Naked | 78 | 18 | 9 | 105 | 27 | 285 |
| Bovine | Sc | 2 | Bovine V. Naked | Naked | 0 | 85 | 12 | 97 | 97 | 192 |
| Bovine | Sc | 3 | Bovine V. Naked | Naked | 59 | 12 | 24 | 95 | 36 | 192 |
| Bovine | Sc | 4 | Bovine V. Naked | Naked | 5 | 73 | 30 | 108 | 103 | 214 |
| Bovine | Sc | 5 | Bovine V. Naked | Naked | 1 | 91 | 14 | 106 | 105 | 214 |
| Bovine | Sc | 1 | Equine V. Bovine | Bovine | 12 | 29 | 28 | 69 | 57 | 173 |
| Bovine | Sc | 2 | Equine V. Bovine | Bovine | 12 | 55 | 37 | 104 | 92 | 173 |
| Bovine | Sc | 3 | Equine V. Bovine | Bovine | 27 | 19 | 35 | 81 | 54 | 202 |
| Bovine | Sc | 4 | Equine V. Bovine | Bovine | 18 | 67 | 36 | 121 | 103 | 202 |
| Bovine | Sc | 5 | Equine V. Bovine | Bovine | 11 | 65 | 31 | 107 | 96 | 151 |
| Bovine | Sc | 1 | Equine V. Bovine | Equine | 1 | 5 | 38 | 44 | 43 | 151 |
| Bovine | Sc | 2 | Equine V. Bovine | Equine | 0 | 6 | 61 | 67 | 67 | 157 |
| Bovine | Sc | 3 | Equine V. Bovine | Equine | 3 | 9 | 78 | 90 | 87 | 157 |
| Bovine | Sc | 4 | Equine V. Bovine | Equine | 5 | 13 | 102 | 120 | 115 | 221 |
| Bovine | Sc | 5 | Equine V. Bovine | Equine | 0 | 32 | 69 | 101 | 101 | 221 |
| Bovine | Sc | 1 | Equine V. Naked | Equine | 3 | 34 | 30 | 67 | 64 | 132 |
| Bovine | Sc | 2 | Equine V. Naked | Equine | 2 | 6 | 57 | 65 | 63 | 132 |
| Bovine | Sc | 3 | Equine V. Naked | Equine | 2 | 13 | 65 | 80 | 78 | 179 |
| Bovine | Sc | 4 | Equine V. Naked | Equine | 2 | 27 | 70 | 99 | 97 | 179 |
| Bovine | Sc | 5 | Equine V. Naked | Equine | 0 | 42 | 58 | 100 | 100 | 200 |
| Bovine | Sc | 1 | Equine V. Naked | Naked | 40 | 33 | 27 | 100 | 60 | 200 |
| Bovine | Sc | 2 | Equine V. Naked | Naked | 0 | 83 | 20 | 103 | 103 | 206 |
| Bovine | Sc | 3 | Equine V. Naked | Naked | 46 | 25 | 32 | 103 | 57 | 206 |
| Bovine | Sc | 4 | Equine V. Naked | Naked | 5 | 80 | 12 | 97 | 92 | 209 |
| Bovine | Sc | 5 | Equine V. Naked | Naked | 6 | 104 | 2 | 112 | 106 | 209 |
| Bovine | Sc | 1 | Naked V. Naked | Control | 61 | 18 | 15 | 94 | 33 | 193 |
| Bovine | Sc | 1 | Naked V. Naked | Control | 77 | 14 | 8 | 99 | 22 | 193 |
| Bovine | Sc | 2 | Naked V. Naked | Control | 3 | 86 | 10 | 99 | 96 | 191 |
| Bovine | Sc | 2 | Naked V. Naked | Control | 1 | 68 | 23 | 92 | 91 | 191 |
| Bovine | Sc | 3 | Naked V. Naked | Control | 62 | 20 | 18 | 100 | 38 | 203 |
| Bovine | Sc | 3 | Naked V. Naked | Control | 32 | 32 | 39 | 103 | 71 | 203 |
| Bovine | Sc | 4 | Naked V. Naked | Control | 6 | 90 | 7 | 103 | 97 | 209 |
| Bovine | Sc | 4 | Naked V. Naked | Control | 5 | 100 | 1 | 106 | 101 | 209 |
| Bovine | Sc | 5 | Naked V. Naked | Control | 5 | 81 | 42 | 128 | 123 | 235 |
| Bovine | Sc | 5 | Naked V. Naked | Control | 2 | 82 | 23 | 107 | 105 | 235 |
| Equine | Sc | 1 | Bovine V. Naked | Bovine | 3 | 14 | 37 | 54 | 51 | 108 |
| Equine | Sc | 2 | Bovine V. Naked | Bovine | 31 | 14 | 9 | 54 | 23 | 108 |
| Equine | Sc | 3 | Bovine V. Naked | Bovine | 7 | 48 | 60 | 115 | 108 | 227 |
| Equine | Sc | 4 | Bovine V. Naked | Bovine | 27 | 40 | 45 | 112 | 85 | 227 |
| Equine | Sc | 5 | Bovine V. Naked | Bovine | 8 | 71 | 40 | 119 | 111 | 209 |
| Equine | Sc | 1 | Bovine V. Naked | Naked | 48 | 36 | 6 | 90 | 42 | 209 |
| Equine | Sc | 2 | Bovine V. Naked | Naked | 67 | 34 | 9 | 110 | 43 | 210 |
| Equine | Sc | 3 | Bovine V. Naked | Naked | 46 | 26 | 28 | 100 | 54 | 210 |
| Equine | Sc | 4 | Bovine V. Naked | Naked | 52 | 33 | 20 | 105 | 53 | 240 |
| Equine | Sc | 5 | Bovine V. Naked | Naked | 82 | 23 | 30 | 135 | 53 | 240 |
| Equine | Sc | 1 | Equine V. Bovine | Bovine | 9 | 20 | 20 | 49 | 40 | 95 |
| Equine | Sc | 2 | Equine V. Bovine | Bovine | 25 | 15 | 6 | 46 | 21 | 95 |
| Equine | Sc | 3 | Equine V. Bovine | Bovine | 25 | 54 | 28 | 107 | 82 | 212 |
| Equine | Sc | 4 | Equine V. Bovine | Bovine | 21 | 42 | 42 | 105 | 84 | 212 |
| Equine | Sc | 5 | Equine V. Bovine | Bovine | 44 | 18 | 68 | 130 | 86 | 214 |
| Equine | Sc | 1 | Equine V. Bovine | Equine | 25 | 11 | 48 | 84 | 59 | 214 |
| Equine | Sc | 2 | Equine V. Bovine | Equine | 6 | 5 | 44 | 55 | 49 | 143 |
| Equine | Sc | 3 | Equine V. Bovine | Equine | 10 | 10 | 68 | 88 | 78 | 143 |
| Equine | Sc | 4 | Equine V. Bovine | Equine | 3 | 16 | 59 | 78 | 75 | 229 |
| Equine | Sc | 5 | Equine V. Bovine | Equine | 8 | 46 | 97 | 151 | 143 | 229 |
| Equine | Sc | 1 | Equine V. Naked | Equine | 6 | 20 | 48 | 74 | 68 | 120 |
| Equine | Sc | 2 | Equine V. Naked | Equine | 9 | 12 | 25 | 46 | 37 | 120 |
| Equine | Sc | 3 | Equine V. Naked | Equine | 4 | 16 | 71 | 91 | 87 | 174 |
| Equine | Sc | 4 | Equine V. Naked | Equine | 4 | 20 | 59 | 83 | 79 | 174 |
| Equine | Sc | 5 | Equine V. Naked | Equine | 3 | 7 | 79 | 89 | 86 | 235 |
| Equine | Sc | 1 | Equine V. Naked | Naked | 57 | 20 | 69 | 146 | 89 | 235 |
| Equine | Sc | 2 | Equine V. Naked | Naked | 6 | 29 | 36 | 71 | 65 | 171 |
| Equine | Sc | 3 | Equine V. Naked | Naked | 34 | 24 | 42 | 100 | 66 | 171 |
| Equine | Sc | 4 | Equine V. Naked | Naked | 71 | 11 | 19 | 101 | 30 | 213 |
| Equine | Sc | 5 | Equine V. Naked | Naked | 54 | 58 | 0 | 112 | 58 | 213 |
| Equine | Sc | 1 | Naked V. Naked | Control | 34 | 40 | 22 | 96 | 62 | 188 |
| Equine | Sc | 1 | Naked V. Naked | Control | 25 | 48 | 19 | 92 | 67 | 188 |
| Equine | Sc | 2 | Naked V. Naked | Control | 70 | 22 | 13 | 105 | 35 | 210 |
| Equine | Sc | 2 | Naked V. Naked | Control | 31 | 36 | 38 | 105 | 74 | 210 |
| Equine | Sc | 3 | Naked V. Naked | Control | 12 | 51 | 41 | 104 | 92 | 214 |
| Equine | Sc | 3 | Naked V. Naked | Control | 77 | 24 | 9 | 110 | 33 | 214 |
| Equine | Sc | 4 | Naked V. Naked | Control | 17 | 53 | 32 | 102 | 85 | 210 |
| Equine | Sc | 4 | Naked V. Naked | Control | 12 | 62 | 34 | 108 | 96 | 210 |
| Equine | Sc | 5 | Naked V. Naked | Control | 77 | 9 | 19 | 105 | 28 | 214 |
| Equine | Sc | 5 | Naked V. Naked | Control | 51 | 22 | 36 | 109 | 58 | 214 |
| Naked | Sc | 1 | Bovine V. Naked | Bovine | 22 | 33 | 20 | 75 | 53 | 167 |
| Naked | Sc | 2 | Bovine V. Naked | Bovine | 6 | 81 | 5 | 92 | 86 | 167 |
| Naked | Sc | 3 | Bovine V. Naked | Bovine | 18 | 56 | 33 | 107 | 89 | 226 |
| Naked | Sc | 4 | Bovine V. Naked | Bovine | 4 | 61 | 54 | 119 | 115 | 226 |
| Naked | Sc | 5 | Bovine V. Naked | Bovine | 7 | 22 | 80 | 109 | 102 | 210 |
| Naked | Sc | 1 | Bovine V. Naked | Naked | 22 | 73 | 6 | 101 | 79 | 210 |
| Naked | Sc | 2 | Bovine V. Naked | Naked | 11 | 90 | 1 | 102 | 91 | 192 |
| Naked | Sc | 3 | Bovine V. Naked | Naked | 2 | 78 | 10 | 90 | 88 | 192 |
| Naked | Sc | 4 | Bovine V. Naked | Naked | 4 | 63 | 36 | 103 | 99 | 206 |
| Naked | Sc | 5 | Bovine V. Naked | Naked | 99 | 4 | 0 | 103 | 4 | 206 |
| Naked | Sc | 1 | Equine V. Bovine | Bovine | 32 | 38 | 21 | 91 | 59 | 193 |
| Naked | Sc | 2 | Equine V. Bovine | Bovine | 5 | 73 | 24 | 102 | 97 | 193 |
| Naked | Sc | 3 | Equine V. Bovine | Bovine | 20 | 40 | 16 | 76 | 56 | 203 |
| Naked | Sc | 4 | Equine V. Bovine | Bovine | 43 | 46 | 38 | 127 | 84 | 203 |
| Naked | Sc | 5 | Equine V. Bovine | Bovine | 50 | 27 | 41 | 118 | 68 | 193 |
| Naked | Sc | 1 | Equine V. Bovine | Equine | 11 | 22 | 42 | 75 | 64 | 193 |
| Naked | Sc | 2 | Equine V. Bovine | Equine | 7 | 41 | 78 | 126 | 119 | 232 |
| Naked | Sc | 3 | Equine V. Bovine | Equine | 17 | 41 | 48 | 106 | 89 | 232 |
| Naked | Sc | 4 | Equine V. Bovine | Equine | 4 | 16 | 75 | 95 | 91 | 214 |
| Naked | Sc | 5 | Equine V. Bovine | Equine | 44 | 36 | 39 | 119 | 75 | 214 |
| Naked | Sc | 1 | Equine V. Naked | Equine | 36 | 44 | 38 | 118 | 82 | 257 |
| Naked | Sc | 2 | Equine V. Naked | Equine | 8 | 39 | 92 | 139 | 131 | 257 |
| Naked | Sc | 3 | Equine V. Naked | Equine | 0 | 12 | 48 | 60 | 60 | 163 |
| Naked | Sc | 4 | Equine V. Naked | Equine | 4 | 9 | 90 | 103 | 99 | 163 |
| Naked | Sc | 5 | Equine V. Naked | Equine | 13 | 11 | 79 | 103 | 90 | 216 |
| Naked | Sc | 1 | Equine V. Naked | Naked | 23 | 89 | 1 | 113 | 90 | 216 |
| Naked | Sc | 2 | Equine V. Naked | Naked | 5 | 89 | 1 | 95 | 90 | 189 |
| Naked | Sc | 3 | Equine V. Naked | Naked | 6 | 83 | 5 | 94 | 88 | 189 |
| Naked | Sc | 4 | Equine V. Naked | Naked | 15 | 71 | 9 | 95 | 80 | 209 |
| Naked | Sc | 5 | Equine V. Naked | Naked | 107 | 7 | 0 | 114 | 7 | 209 |
| Naked | Sc | 1 | Naked V. Naked | Control | 14 | 67 | 21 | 102 | 88 | 197 |
| Naked | Sc | 1 | Naked V. Naked | Control | 9 | 68 | 18 | 95 | 86 | 197 |
| Naked | Sc | 2 | Naked V. Naked | Control | 6 | 88 | 5 | 99 | 93 | 193 |
| Naked | Sc | 2 | Naked V. Naked | Control | 2 | 68 | 24 | 94 | 92 | 193 |
| Naked | Sc | 3 | Naked V. Naked | Control | 5 | 61 | 22 | 88 | 83 | 202 |
| Naked | Sc | 3 | Naked V. Naked | Control | 4 | 82 | 28 | 114 | 110 | 202 |
| Naked | Sc | 4 | Naked V. Naked | Control | 8 | 71 | 25 | 104 | 96 | 210 |
| Naked | Sc | 4 | Naked V. Naked | Control | 7 | 83 | 16 | 106 | 99 | 210 |
| Naked | Sc | 5 | Naked V. Naked | Control | 82 | 17 | 15 | 114 | 32 | 222 |
| Naked | Sc | 5 | Naked V. Naked | Control | 48 | 19 | 41 | 108 | 60 | 222 |
| Parasitoid Treatment | Species | Treatment Number | Arena | Side | Adult | Uneclosed | Parasitoid | Total Pupae | Total Uneclosed pupae in treatment in plate | Total Uneclosed pupae in cage |
| Bovine | Mr | 1 | Bovine V. Naked | Bovine | 31 | 29 | 66 | 126 | 95 | 275 |
| Bovine | Mr | 2 | Bovine V. Naked | Bovine | 110 | 28 | 11 | 149 | 39 | 275 |
| Bovine | Mr | 3 | Bovine V. Naked | Bovine | 82 | 11 | 22 | 115 | 33 | 259 |
| Bovine | Mr | 4 | Bovine V. Naked | Bovine | 95 | 26 | 23 | 144 | 49 | 259 |
| Bovine | Mr | 5 | Bovine V. Naked | Bovine | 57 | 29 | 29 | 115 | 58 | 218 |
| Bovine | Mr | 1 | Bovine V. Naked | Naked | 61 | 16 | 26 | 103 | 42 | 218 |
| Bovine | Mr | 2 | Bovine V. Naked | Naked | 39 | 22 | 44 | 105 | 66 | 208 |
| Bovine | Mr | 3 | Bovine V. Naked | Naked | 93 | 7 | 3 | 103 | 10 | 208 |
| Bovine | Mr | 4 | Bovine V. Naked | Naked | 41 | 14 | 49 | 104 | 63 | 209 |
| Bovine | Mr | 5 | Bovine V. Naked | Naked | 4 | 10 | 91 | 105 | 101 | 209 |
| Bovine | Mr | 1 | Equine V. Bovine | Bovine | 75 | 17 | 52 | 144 | 69 | 262 |
| Bovine | Mr | 2 | Equine V. Bovine | Bovine | 69 | 2 | 47 | 118 | 49 | 262 |
| Bovine | Mr | 3 | Equine V. Bovine | Bovine | 69 | 9 | 37 | 115 | 46 | 259 |
| Bovine | Mr | 4 | Equine V. Bovine | Bovine | 81 | 7 | 56 | 144 | 63 | 259 |
| Bovine | Mr | 5 | Equine V. Bovine | Bovine | 43 | 12 | 39 | 94 | 51 | 194 |
| Bovine | Mr | 1 | Equine V. Bovine | Equine | 65 | 11 | 24 | 100 | 35 | 194 |
| Bovine | Mr | 2 | Equine V. Bovine | Equine | 61 | 15 | 26 | 102 | 41 | 229 |
| Bovine | Mr | 3 | Equine V. Bovine | Equine | 74 | 34 | 19 | 127 | 53 | 229 |
| Bovine | Mr | 4 | Equine V. Bovine | Equine | 89 | 11 | 4 | 104 | 15 | 181 |
| Bovine | Mr | 5 | Equine V. Bovine | Equine | 52 | 15 | 10 | 77 | 25 | 181 |
| Bovine | Mr | 1 | Equine V. Naked | Equine | 38 | 15 | 54 | 107 | 69 | 211 |
| Bovine | Mr | 2 | Equine V. Naked | Equine | 81 | 5 | 18 | 104 | 23 | 211 |
| Bovine | Mr | 3 | Equine V. Naked | Equine | 65 | 9 | 34 | 108 | 43 | 220 |
| Bovine | Mr | 4 | Equine V. Naked | Equine | 79 | 18 | 15 | 112 | 33 | 220 |
| Bovine | Mr | 5 | Equine V. Naked | Equine | 58 | 12 | 22 | 92 | 34 | 199 |
| Bovine | Mr | 1 | Equine V. Naked | Naked | 64 | 17 | 26 | 107 | 43 | 199 |
| Bovine | Mr | 2 | Equine V. Naked | Naked | 84 | 6 | 15 | 105 | 21 | 208 |
| Bovine | Mr | 3 | Equine V. Naked | Naked | 95 | 4 | 4 | 103 | 8 | 208 |
| Bovine | Mr | 4 | Equine V. Naked | Naked | 83 | 12 | 17 | 112 | 29 | 213 |
| Bovine | Mr | 5 | Equine V. Naked | Naked | 6 | 26 | 69 | 101 | 95 | 213 |
| Bovine | Mr | 1 | Naked V. Naked | Control | 1 | 16 | 90 | 107 | 106 | 211 |
| Bovine | Mr | 1 | Naked V. Naked | Control | 42 | 19 | 43 | 104 | 62 | 211 |
| Bovine | Mr | 2 | Naked V. Naked | Control | 71 | 12 | 21 | 104 | 33 | 206 |
| Bovine | Mr | 2 | Naked V. Naked | Control | 72 | 10 | 20 | 102 | 30 | 206 |
| Bovine | Mr | 3 | Naked V. Naked | Control | 97 | 7 | 0 | 104 | 7 | 210 |
| Bovine | Mr | 3 | Naked V. Naked | Control | 47 | 16 | 43 | 106 | 59 | 210 |
| Bovine | Mr | 4 | Naked V. Naked | Control | 21 | 20 | 69 | 110 | 89 | 239 |
| Bovine | Mr | 4 | Naked V. Naked | Control | 55 | 5 | 69 | 129 | 74 | 239 |
| Bovine | Mr | 5 | Naked V. Naked | Control | 12 | 16 | 86 | 114 | 102 | 221 |
| Bovine | Mr | 5 | Naked V. Naked | Control | 6 | 23 | 78 | 107 | 101 | 221 |
| Equine | Mr | 1 | Bovine V. Naked | Bovine | 77 | 4 | 32 | 113 | 36 | 221 |
| Equine | Mr | 2 | Bovine V. Naked | Bovine | 82 | 1 | 25 | 108 | 26 | 221 |
| Equine | Mr | 3 | Bovine V. Naked | Bovine | 49 | 50 | 37 | 136 | 87 | 274 |
| Equine | Mr | 4 | Bovine V. Naked | Bovine | 76 | 18 | 44 | 138 | 62 | 274 |
| Equine | Mr | 5 | Bovine V. Naked | Bovine | 62 | 14 | 24 | 100 | 38 | 212 |
| Equine | Mr | 1 | Bovine V. Naked | Naked | 56 | 18 | 38 | 112 | 56 | 212 |
| Equine | Mr | 2 | Bovine V. Naked | Naked | 100 | 8 | 0 | 108 | 8 | 215 |
| Equine | Mr | 3 | Bovine V. Naked | Naked | 8 | 20 | 79 | 107 | 99 | 215 |
| Equine | Mr | 4 | Bovine V. Naked | Naked | 77 | 13 | 41 | 131 | 54 | 240 |
| Equine | Mr | 5 | Bovine V. Naked | Naked | 39 | 17 | 53 | 109 | 70 | 240 |
| Equine | Mr | 1 | Equine V. Bovine | Bovine | 52 | 21 | 23 | 96 | 44 | 241 |
| Equine | Mr | 2 | Equine V. Bovine | Bovine | 90 | 23 | 32 | 145 | 55 | 241 |
| Equine | Mr | 3 | Equine V. Bovine | Bovine | 60 | 51 | 35 | 146 | 86 | 285 |
| Equine | Mr | 4 | Equine V. Bovine | Bovine | 67 | 36 | 36 | 139 | 72 | 285 |
| Equine | Mr | 5 | Equine V. Bovine | Bovine | 57 | 34 | 26 | 117 | 60 | 210 |
| Equine | Mr | 1 | Equine V. Bovine | Equine | 61 | 6 | 26 | 93 | 32 | 210 |
| Equine | Mr | 2 | Equine V. Bovine | Equine | 64 | 24 | 16 | 104 | 40 | 202 |
| Equine | Mr | 3 | Equine V. Bovine | Equine | 58 | 1 | 39 | 98 | 40 | 202 |
| Equine | Mr | 4 | Equine V. Bovine | Equine | 62 | 3 | 35 | 100 | 38 | 234 |
| Equine | Mr | 5 | Equine V. Bovine | Equine | 108 | 6 | 20 | 134 | 26 | 234 |
| Equine | Mr | 1 | Equine V. Naked | Equine | 72 | 3 | 30 | 105 | 33 | 207 |
| Equine | Mr | 2 | Equine V. Naked | Equine | 57 | 3 | 42 | 102 | 45 | 207 |
| Equine | Mr | 3 | Equine V. Naked | Equine | 60 | 24 | 21 | 105 | 45 | 224 |
| Equine | Mr | 4 | Equine V. Naked | Equine | 68 | 48 | 3 | 119 | 51 | 224 |
| Equine | Mr | 5 | Equine V. Naked | Equine | 68 | 23 | 34 | 125 | 57 | 244 |
| Equine | Mr | 1 | Equine V. Naked | Naked | 90 | 6 | 23 | 119 | 29 | 244 |
| Equine | Mr | 2 | Equine V. Naked | Naked | 52 | 7 | 51 | 110 | 58 | 220 |
| Equine | Mr | 3 | Equine V. Naked | Naked | 66 | 4 | 40 | 110 | 44 | 220 |
| Equine | Mr | 4 | Equine V. Naked | Naked | 88 | 6 | 36 | 130 | 42 | 240 |
| Equine | Mr | 5 | Equine V. Naked | Naked | 64 | 6 | 40 | 110 | 46 | 240 |
| Equine | Mr | 1 | Naked V. Naked | Control | 35 | 29 | 59 | 123 | 88 | 237 |
| Equine | Mr | 1 | Naked V. Naked | Control | 38 | 19 | 57 | 114 | 76 | 237 |
| Equine | Mr | 2 | Naked V. Naked | Control | 66 | 12 | 28 | 106 | 40 | 215 |
| Equine | Mr | 2 | Naked V. Naked | Control | 10 | 6 | 93 | 109 | 99 | 215 |
| Equine | Mr | 3 | Naked V. Naked | Control | 25 | 24 | 63 | 112 | 87 | 218 |
| Equine | Mr | 3 | Naked V. Naked | Control | 25 | 20 | 61 | 106 | 81 | 218 |
| Equine | Mr | 4 | Naked V. Naked | Control | 96 | 11 | 48 | 155 | 59 | 295 |
| Equine | Mr | 4 | Naked V. Naked | Control | 96 | 2 | 42 | 140 | 44 | 295 |
| Equine | Mr | 5 | Naked V. Naked | Control | 8 | 28 | 70 | 106 | 98 | 249 |
| Equine | Mr | 5 | Naked V. Naked | Control | 85 | 12 | 46 | 143 | 58 | 249 |
| Naked | Mr | 1 | Bovine V. Naked | Bovine | 92 | 0 | 40 | 132 | 40 | 266 |
| Naked | Mr | 2 | Bovine V. Naked | Bovine | 88 | 19 | 27 | 134 | 46 | 266 |
| Naked | Mr | 3 | Bovine V. Naked | Bovine | 87 | 5 | 26 | 118 | 31 | 246 |
| Naked | Mr | 4 | Bovine V. Naked | Bovine | 77 | 23 | 28 | 128 | 51 | 246 |
| Naked | Mr | 5 | Bovine V. Naked | Bovine | 49 | 23 | 27 | 99 | 50 | 201 |
| Naked | Mr | 1 | Bovine V. Naked | Naked | 100 | 2 | 0 | 102 | 2 | 201 |
| Naked | Mr | 2 | Bovine V. Naked | Naked | 26 | 25 | 48 | 99 | 73 | 213 |
| Naked | Mr | 3 | Bovine V. Naked | Naked | 24 | 19 | 71 | 114 | 90 | 213 |
| Naked | Mr | 4 | Bovine V. Naked | Naked | 37 | 11 | 56 | 104 | 67 | 203 |
| Naked | Mr | 5 | Bovine V. Naked | Naked | 2 | 20 | 77 | 99 | 97 | 203 |
| Naked | Mr | 1 | Equine V. Bovine | Bovine | 77 | 9 | 48 | 134 | 57 | 268 |
| Naked | Mr | 2 | Equine V. Bovine | Bovine | 55 | 29 | 50 | 134 | 79 | 268 |
| Naked | Mr | 3 | Equine V. Bovine | Bovine | 75 | 17 | 24 | 116 | 41 | 232 |
| Naked | Mr | 4 | Equine V. Bovine | Bovine | 46 | 20 | 50 | 116 | 70 | 232 |
| Naked | Mr | 5 | Equine V. Bovine | Bovine | 17 | 33 | 43 | 93 | 76 | 200 |
| Naked | Mr | 1 | Equine V. Bovine | Equine | 76 | 0 | 31 | 107 | 31 | 200 |
| Naked | Mr | 2 | Equine V. Bovine | Equine | 71 | 12 | 32 | 115 | 44 | 224 |
| Naked | Mr | 3 | Equine V. Bovine | Equine | 62 | 31 | 16 | 109 | 47 | 224 |
| Naked | Mr | 4 | Equine V. Bovine | Equine | 79 | 8 | 26 | 113 | 34 | 234 |
| Naked | Mr | 5 | Equine V. Bovine | Equine | 77 | 22 | 22 | 121 | 44 | 234 |
| Naked | Mr | 1 | Equine V. Naked | Equine | 72 | 15 | 26 | 113 | 41 | 245 |
| Naked | Mr | 2 | Equine V. Naked | Equine | 76 | 20 | 36 | 132 | 56 | 245 |
| Naked | Mr | 3 | Equine V. Naked | Equine | 67 | 13 | 19 | 99 | 32 | 212 |
| Naked | Mr | 4 | Equine V. Naked | Equine | 81 | 9 | 23 | 113 | 32 | 212 |
| Naked | Mr | 5 | Equine V. Naked | Equine | 76 | 14 | 6 | 96 | 20 | 232 |
| Naked | Mr | 1 | Equine V. Naked | Naked | 80 | 6 | 50 | 136 | 56 | 232 |
| Naked | Mr | 2 | Equine V. Naked | Naked | 7 | 23 | 73 | 103 | 96 | 208 |
| Naked | Mr | 3 | Equine V. Naked | Naked | 2 | 22 | 81 | 105 | 103 | 208 |
| Naked | Mr | 4 | Equine V. Naked | Naked | 3 | 20 | 81 | 104 | 101 | 204 |
| Naked | Mr | 5 | Equine V. Naked | Naked | 7 | 20 | 73 | 100 | 93 | 204 |
| Naked | Mr | 1 | Naked V. Naked | Control | 36 | 6 | 90 | 132 | 96 | 262 |
| Naked | Mr | 1 | Naked V. Naked | Control | 90 | 8 | 32 | 130 | 38 | 262 |
| Naked | Mr | 2 | Naked V. Naked | Control | 3 | 17 | 85 | 105 | 102 | 221 |
| Naked | Mr | 2 | Naked V. Naked | Control | 40 | 23 | 53 | 116 | 76 | 221 |
| Naked | Mr | 3 | Naked V. Naked | Control | 39 | 13 | 66 | 118 | 79 | 227 |
| Naked | Mr | 3 | Naked V. Naked | Control | 3 | 35 | 71 | 109 | 106 | 227 |
| Naked | Mr | 4 | Naked V. Naked | Control | 7 | 18 | 84 | 109 | 102 | 219 |
| Naked | Mr | 4 | Naked V. Naked | Control | 28 | 18 | 64 | 110 | 82 | 219 |
| Naked | Mr | 5 | Naked V. Naked | Control | 0 | 27 | 88 | 115 | 115 | 215 |
| Naked | Mr | 5 | Naked V. Naked | Control | 5 | 16 | 79 | 100 | 95 | 215 |
| Bovine | Sc | 1 | Bovine V. Naked | Bovine | 2 | 23 | 21 | 46 | 53 | 186 |
| Bovine | Sc | 2 | Bovine V. Naked | Bovine | 14 | 86 | 40 | 140 | 100 | 186 |
| Bovine | Sc | 3 | Bovine V. Naked | Bovine | 18 | 21 | 25 | 64 | 49 | 207 |
| Bovine | Sc | 4 | Bovine V. Naked | Bovine | 5 | 76 | 62 | 143 | 113 | 207 |
| Bovine | Sc | 5 | Bovine V. Naked | Bovine | 8 | 89 | 83 | 180 | 124 | 285 |
| Bovine | Sc | 1 | Bovine V. Naked | Naked | 78 | 18 | 9 | 105 | 27 | 285 |
| Bovine | Sc | 2 | Bovine V. Naked | Naked | 0 | 85 | 12 | 97 | 97 | 192 |
| Bovine | Sc | 3 | Bovine V. Naked | Naked | 59 | 12 | 24 | 95 | 36 | 192 |
| Bovine | Sc | 4 | Bovine V. Naked | Naked | 5 | 73 | 30 | 108 | 103 | 214 |
| Bovine | Sc | 5 | Bovine V. Naked | Naked | 1 | 91 | 14 | 106 | 105 | 214 |
| Bovine | Sc | 1 | Equine V. Bovine | Bovine | 12 | 29 | 28 | 69 | 57 | 173 |
| Bovine | Sc | 2 | Equine V. Bovine | Bovine | 12 | 55 | 37 | 104 | 92 | 173 |
| Bovine | Sc | 3 | Equine V. Bovine | Bovine | 27 | 19 | 35 | 81 | 54 | 202 |
| Bovine | Sc | 4 | Equine V. Bovine | Bovine | 18 | 67 | 36 | 121 | 103 | 202 |
| Bovine | Sc | 5 | Equine V. Bovine | Bovine | 11 | 65 | 31 | 107 | 96 | 151 |
| Bovine | Sc | 1 | Equine V. Bovine | Equine | 1 | 5 | 38 | 44 | 43 | 151 |
| Bovine | Sc | 2 | Equine V. Bovine | Equine | 0 | 6 | 61 | 67 | 67 | 157 |
| Bovine | Sc | 3 | Equine V. Bovine | Equine | 3 | 9 | 78 | 90 | 87 | 157 |
| Bovine | Sc | 4 | Equine V. Bovine | Equine | 5 | 13 | 102 | 120 | 115 | 221 |
| Bovine | Sc | 5 | Equine V. Bovine | Equine | 0 | 32 | 69 | 101 | 101 | 221 |
| Bovine | Sc | 1 | Equine V. Naked | Equine | 3 | 34 | 30 | 67 | 64 | 132 |
| Bovine | Sc | 2 | Equine V. Naked | Equine | 2 | 6 | 57 | 65 | 63 | 132 |
| Bovine | Sc | 3 | Equine V. Naked | Equine | 2 | 13 | 65 | 80 | 78 | 179 |
| Bovine | Sc | 4 | Equine V. Naked | Equine | 2 | 27 | 70 | 99 | 97 | 179 |
| Bovine | Sc | 5 | Equine V. Naked | Equine | 0 | 42 | 58 | 100 | 100 | 200 |
| Bovine | Sc | 1 | Equine V. Naked | Naked | 40 | 33 | 27 | 100 | 60 | 200 |
| Bovine | Sc | 2 | Equine V. Naked | Naked | 0 | 83 | 20 | 103 | 103 | 206 |
| Bovine | Sc | 3 | Equine V. Naked | Naked | 46 | 25 | 32 | 103 | 57 | 206 |
| Bovine | Sc | 4 | Equine V. Naked | Naked | 5 | 80 | 12 | 97 | 92 | 209 |
| Bovine | Sc | 5 | Equine V. Naked | Naked | 6 | 104 | 2 | 112 | 106 | 209 |
| Bovine | Sc | 1 | Naked V. Naked | Control | 61 | 18 | 15 | 94 | 33 | 193 |
| Bovine | Sc | 1 | Naked V. Naked | Control | 77 | 14 | 8 | 99 | 22 | 193 |
| Bovine | Sc | 2 | Naked V. Naked | Control | 3 | 86 | 10 | 99 | 96 | 191 |
| Bovine | Sc | 2 | Naked V. Naked | Control | 1 | 68 | 23 | 92 | 91 | 191 |
| Bovine | Sc | 3 | Naked V. Naked | Control | 62 | 20 | 18 | 100 | 38 | 203 |
| Bovine | Sc | 3 | Naked V. Naked | Control | 32 | 32 | 39 | 103 | 71 | 203 |
| Bovine | Sc | 4 | Naked V. Naked | Control | 6 | 90 | 7 | 103 | 97 | 209 |
| Bovine | Sc | 4 | Naked V. Naked | Control | 5 | 100 | 1 | 106 | 101 | 209 |
| Bovine | Sc | 5 | Naked V. Naked | Control | 5 | 81 | 42 | 128 | 123 | 235 |
| Bovine | Sc | 5 | Naked V. Naked | Control | 2 | 82 | 23 | 107 | 105 | 235 |
| Equine | Sc | 1 | Bovine V. Naked | Bovine | 3 | 14 | 37 | 54 | 51 | 108 |
| Equine | Sc | 2 | Bovine V. Naked | Bovine | 31 | 14 | 9 | 54 | 23 | 108 |
| Equine | Sc | 3 | Bovine V. Naked | Bovine | 7 | 48 | 60 | 115 | 108 | 227 |
| Equine | Sc | 4 | Bovine V. Naked | Bovine | 27 | 40 | 45 | 112 | 85 | 227 |
| Equine | Sc | 5 | Bovine V. Naked | Bovine | 8 | 71 | 40 | 119 | 111 | 209 |
| Equine | Sc | 1 | Bovine V. Naked | Naked | 48 | 36 | 6 | 90 | 42 | 209 |
| Equine | Sc | 2 | Bovine V. Naked | Naked | 67 | 34 | 9 | 110 | 43 | 210 |
| Equine | Sc | 3 | Bovine V. Naked | Naked | 46 | 26 | 28 | 100 | 54 | 210 |
| Equine | Sc | 4 | Bovine V. Naked | Naked | 52 | 33 | 20 | 105 | 53 | 240 |
| Equine | Sc | 5 | Bovine V. Naked | Naked | 82 | 23 | 30 | 135 | 53 | 240 |
| Equine | Sc | 1 | Equine V. Bovine | Bovine | 9 | 20 | 20 | 49 | 40 | 95 |
| Equine | Sc | 2 | Equine V. Bovine | Bovine | 25 | 15 | 6 | 46 | 21 | 95 |
| Equine | Sc | 3 | Equine V. Bovine | Bovine | 25 | 54 | 28 | 107 | 82 | 212 |
| Equine | Sc | 4 | Equine V. Bovine | Bovine | 21 | 42 | 42 | 105 | 84 | 212 |
| Equine | Sc | 5 | Equine V. Bovine | Bovine | 44 | 18 | 68 | 130 | 86 | 214 |
| Equine | Sc | 1 | Equine V. Bovine | Equine | 25 | 11 | 48 | 84 | 59 | 214 |
| Equine | Sc | 2 | Equine V. Bovine | Equine | 6 | 5 | 44 | 55 | 49 | 143 |
| Equine | Sc | 3 | Equine V. Bovine | Equine | 10 | 10 | 68 | 88 | 78 | 143 |
| Equine | Sc | 4 | Equine V. Bovine | Equine | 3 | 16 | 59 | 78 | 75 | 229 |
| Equine | Sc | 5 | Equine V. Bovine | Equine | 8 | 46 | 97 | 151 | 143 | 229 |
| Equine | Sc | 1 | Equine V. Naked | Equine | 6 | 20 | 48 | 74 | 68 | 120 |
| Equine | Sc | 2 | Equine V. Naked | Equine | 9 | 12 | 25 | 46 | 37 | 120 |
| Equine | Sc | 3 | Equine V. Naked | Equine | 4 | 16 | 71 | 91 | 87 | 174 |
| Equine | Sc | 4 | Equine V. Naked | Equine | 4 | 20 | 59 | 83 | 79 | 174 |
| Equine | Sc | 5 | Equine V. Naked | Equine | 3 | 7 | 79 | 89 | 86 | 235 |
| Equine | Sc | 1 | Equine V. Naked | Naked | 57 | 20 | 69 | 146 | 89 | 235 |
| Equine | Sc | 2 | Equine V. Naked | Naked | 6 | 29 | 36 | 71 | 65 | 171 |
| Equine | Sc | 3 | Equine V. Naked | Naked | 34 | 24 | 42 | 100 | 66 | 171 |
| Equine | Sc | 4 | Equine V. Naked | Naked | 71 | 11 | 19 | 101 | 30 | 213 |
| Equine | Sc | 5 | Equine V. Naked | Naked | 54 | 58 | 0 | 112 | 58 | 213 |
| Equine | Sc | 1 | Naked V. Naked | Control | 34 | 40 | 22 | 96 | 62 | 188 |
| Equine | Sc | 1 | Naked V. Naked | Control | 25 | 48 | 19 | 92 | 67 | 188 |
| Equine | Sc | 2 | Naked V. Naked | Control | 70 | 22 | 13 | 105 | 35 | 210 |
| Equine | Sc | 2 | Naked V. Naked | Control | 31 | 36 | 38 | 105 | 74 | 210 |
| Equine | Sc | 3 | Naked V. Naked | Control | 12 | 51 | 41 | 104 | 92 | 214 |
| Equine | Sc | 3 | Naked V. Naked | Control | 77 | 24 | 9 | 110 | 33 | 214 |
| Equine | Sc | 4 | Naked V. Naked | Control | 17 | 53 | 32 | 102 | 85 | 210 |
| Equine | Sc | 4 | Naked V. Naked | Control | 12 | 62 | 34 | 108 | 96 | 210 |
| Equine | Sc | 5 | Naked V. Naked | Control | 77 | 9 | 19 | 105 | 28 | 214 |
| Equine | Sc | 5 | Naked V. Naked | Control | 51 | 22 | 36 | 109 | 58 | 214 |
| Naked | Sc | 1 | Bovine V. Naked | Bovine | 22 | 33 | 20 | 75 | 53 | 167 |
| Naked | Sc | 2 | Bovine V. Naked | Bovine | 6 | 81 | 5 | 92 | 86 | 167 |
| Naked | Sc | 3 | Bovine V. Naked | Bovine | 18 | 56 | 33 | 107 | 89 | 226 |
| Naked | Sc | 4 | Bovine V. Naked | Bovine | 4 | 61 | 54 | 119 | 115 | 226 |
| Naked | Sc | 5 | Bovine V. Naked | Bovine | 7 | 22 | 80 | 109 | 102 | 210 |
| Naked | Sc | 1 | Bovine V. Naked | Naked | 22 | 73 | 6 | 101 | 79 | 210 |
| Naked | Sc | 2 | Bovine V. Naked | Naked | 11 | 90 | 1 | 102 | 91 | 192 |
| Naked | Sc | 3 | Bovine V. Naked | Naked | 2 | 78 | 10 | 90 | 88 | 192 |
| Naked | Sc | 4 | Bovine V. Naked | Naked | 4 | 63 | 36 | 103 | 99 | 206 |
| Naked | Sc | 5 | Bovine V. Naked | Naked | 99 | 4 | 0 | 103 | 4 | 206 |
| Naked | Sc | 1 | Equine V. Bovine | Bovine | 32 | 38 | 21 | 91 | 59 | 193 |
| Naked | Sc | 2 | Equine V. Bovine | Bovine | 5 | 73 | 24 | 102 | 97 | 193 |
| Naked | Sc | 3 | Equine V. Bovine | Bovine | 20 | 40 | 16 | 76 | 56 | 203 |
| Naked | Sc | 4 | Equine V. Bovine | Bovine | 43 | 46 | 38 | 127 | 84 | 203 |
| Naked | Sc | 5 | Equine V. Bovine | Bovine | 50 | 27 | 41 | 118 | 68 | 193 |
| Naked | Sc | 1 | Equine V. Bovine | Equine | 11 | 22 | 42 | 75 | 64 | 193 |
| Naked | Sc | 2 | Equine V. Bovine | Equine | 7 | 41 | 78 | 126 | 119 | 232 |
| Naked | Sc | 3 | Equine V. Bovine | Equine | 17 | 41 | 48 | 106 | 89 | 232 |
| Naked | Sc | 4 | Equine V. Bovine | Equine | 4 | 16 | 75 | 95 | 91 | 214 |
| Naked | Sc | 5 | Equine V. Bovine | Equine | 44 | 36 | 39 | 119 | 75 | 214 |
| Naked | Sc | 1 | Equine V. Naked | Equine | 36 | 44 | 38 | 118 | 82 | 257 |
| Naked | Sc | 2 | Equine V. Naked | Equine | 8 | 39 | 92 | 139 | 131 | 257 |
| Naked | Sc | 3 | Equine V. Naked | Equine | 0 | 12 | 48 | 60 | 60 | 163 |
| Naked | Sc | 4 | Equine V. Naked | Equine | 4 | 9 | 90 | 103 | 99 | 163 |
| Naked | Sc | 5 | Equine V. Naked | Equine | 13 | 11 | 79 | 103 | 90 | 216 |
| Naked | Sc | 1 | Equine V. Naked | Naked | 23 | 89 | 1 | 113 | 90 | 216 |
| Naked | Sc | 2 | Equine V. Naked | Naked | 5 | 89 | 1 | 95 | 90 | 189 |
| Naked | Sc | 3 | Equine V. Naked | Naked | 6 | 83 | 5 | 94 | 88 | 189 |
| Naked | Sc | 4 | Equine V. Naked | Naked | 15 | 71 | 9 | 95 | 80 | 209 |
| Naked | Sc | 5 | Equine V. Naked | Naked | 107 | 7 | 0 | 114 | 7 | 209 |
| Naked | Sc | 1 | Naked V. Naked | Control | 14 | 67 | 21 | 102 | 88 | 197 |
| Naked | Sc | 1 | Naked V. Naked | Control | 9 | 68 | 18 | 95 | 86 | 197 |
| Naked | Sc | 2 | Naked V. Naked | Control | 6 | 88 | 5 | 99 | 93 | 193 |
| Naked | Sc | 2 | Naked V. Naked | Control | 2 | 68 | 24 | 94 | 92 | 193 |
| Naked | Sc | 3 | Naked V. Naked | Control | 5 | 61 | 22 | 88 | 83 | 202 |
| Naked | Sc | 3 | Naked V. Naked | Control | 4 | 82 | 28 | 114 | 110 | 202 |
| Naked | Sc | 4 | Naked V. Naked | Control | 8 | 71 | 25 | 104 | 96 | 210 |
| Naked | Sc | 4 | Naked V. Naked | Control | 7 | 83 | 16 | 106 | 99 | 210 |
| Naked | Sc | 5 | Naked V. Naked | Control | 82 | 17 | 15 | 114 | 32 | 222 |
| Naked | Sc | 5 | Naked V. Naked | Control | 48 | 19 | 41 | 108 | 60 | 222 |
| Parasitoid Treatment | Species | Treatment Number | Arena | Side | Adult | Uneclosed | Parasitoid | Total Pupae | Total Uneclosed pupae in treatment in plate | Total Uneclosed pupae in cage |
| Bovine | Mr | 1 | Bovine V. Naked | Bovine | 31 | 29 | 66 | 126 | 95 | 275 |
| Bovine | Mr | 2 | Bovine V. Naked | Bovine | 110 | 28 | 11 | 149 | 39 | 275 |
| Bovine | Mr | 3 | Bovine V. Naked | Bovine | 82 | 11 | 22 | 115 | 33 | 259 |
| Bovine | Mr | 4 | Bovine V. Naked | Bovine | 95 | 26 | 23 | 144 | 49 | 259 |
| Bovine | Mr | 5 | Bovine V. Naked | Bovine | 57 | 29 | 29 | 115 | 58 | 218 |
| Bovine | Mr | 1 | Bovine V. Naked | Naked | 61 | 16 | 26 | 103 | 42 | 218 |
| Bovine | Mr | 2 | Bovine V. Naked | Naked | 39 | 22 | 44 | 105 | 66 | 208 |
| Bovine | Mr | 3 | Bovine V. Naked | Naked | 93 | 7 | 3 | 103 | 10 | 208 |
| Bovine | Mr | 4 | Bovine V. Naked | Naked | 41 | 14 | 49 | 104 | 63 | 209 |
| Bovine | Mr | 5 | Bovine V. Naked | Naked | 4 | 10 | 91 | 105 | 101 | 209 |
| Bovine | Mr | 1 | Equine V. Bovine | Bovine | 75 | 17 | 52 | 144 | 69 | 262 |
| Bovine | Mr | 2 | Equine V. Bovine | Bovine | 69 | 2 | 47 | 118 | 49 | 262 |
| Bovine | Mr | 3 | Equine V. Bovine | Bovine | 69 | 9 | 37 | 115 | 46 | 259 |
| Bovine | Mr | 4 | Equine V. Bovine | Bovine | 81 | 7 | 56 | 144 | 63 | 259 |
| Bovine | Mr | 5 | Equine V. Bovine | Bovine | 43 | 12 | 39 | 94 | 51 | 194 |
| Bovine | Mr | 1 | Equine V. Bovine | Equine | 65 | 11 | 24 | 100 | 35 | 194 |
| Bovine | Mr | 2 | Equine V. Bovine | Equine | 61 | 15 | 26 | 102 | 41 | 229 |
| Bovine | Mr | 3 | Equine V. Bovine | Equine | 74 | 34 | 19 | 127 | 53 | 229 |
| Bovine | Mr | 4 | Equine V. Bovine | Equine | 89 | 11 | 4 | 104 | 15 | 181 |
| Bovine | Mr | 5 | Equine V. Bovine | Equine | 52 | 15 | 10 | 77 | 25 | 181 |
| Bovine | Mr | 1 | Equine V. Naked | Equine | 38 | 15 | 54 | 107 | 69 | 211 |
| Bovine | Mr | 2 | Equine V. Naked | Equine | 81 | 5 | 18 | 104 | 23 | 211 |
| Bovine | Mr | 3 | Equine V. Naked | Equine | 65 | 9 | 34 | 108 | 43 | 220 |
| Bovine | Mr | 4 | Equine V. Naked | Equine | 79 | 18 | 15 | 112 | 33 | 220 |
| Bovine | Mr | 5 | Equine V. Naked | Equine | 58 | 12 | 22 | 92 | 34 | 199 |
| Bovine | Mr | 1 | Equine V. Naked | Naked | 64 | 17 | 26 | 107 | 43 | 199 |
| Bovine | Mr | 2 | Equine V. Naked | Naked | 84 | 6 | 15 | 105 | 21 | 208 |
| Bovine | Mr | 3 | Equine V. Naked | Naked | 95 | 4 | 4 | 103 | 8 | 208 |
| Bovine | Mr | 4 | Equine V. Naked | Naked | 83 | 12 | 17 | 112 | 29 | 213 |
| Bovine | Mr | 5 | Equine V. Naked | Naked | 6 | 26 | 69 | 101 | 95 | 213 |
| Bovine | Mr | 1 | Naked V. Naked | Control | 1 | 16 | 90 | 107 | 106 | 211 |
| Bovine | Mr | 1 | Naked V. Naked | Control | 42 | 19 | 43 | 104 | 62 | 211 |
| Bovine | Mr | 2 | Naked V. Naked | Control | 71 | 12 | 21 | 104 | 33 | 206 |
| Bovine | Mr | 2 | Naked V. Naked | Control | 72 | 10 | 20 | 102 | 30 | 206 |
| Bovine | Mr | 3 | Naked V. Naked | Control | 97 | 7 | 0 | 104 | 7 | 210 |
| Bovine | Mr | 3 | Naked V. Naked | Control | 47 | 16 | 43 | 106 | 59 | 210 |
| Bovine | Mr | 4 | Naked V. Naked | Control | 21 | 20 | 69 | 110 | 89 | 239 |
| Bovine | Mr | 4 | Naked V. Naked | Control | 55 | 5 | 69 | 129 | 74 | 239 |
| Bovine | Mr | 5 | Naked V. Naked | Control | 12 | 16 | 86 | 114 | 102 | 221 |
| Bovine | Mr | 5 | Naked V. Naked | Control | 6 | 23 | 78 | 107 | 101 | 221 |
| Equine | Mr | 1 | Bovine V. Naked | Bovine | 77 | 4 | 32 | 113 | 36 | 221 |
| Equine | Mr | 2 | Bovine V. Naked | Bovine | 82 | 1 | 25 | 108 | 26 | 221 |
| Equine | Mr | 3 | Bovine V. Naked | Bovine | 49 | 50 | 37 | 136 | 87 | 274 |
| Equine | Mr | 4 | Bovine V. Naked | Bovine | 76 | 18 | 44 | 138 | 62 | 274 |
| Equine | Mr | 5 | Bovine V. Naked | Bovine | 62 | 14 | 24 | 100 | 38 | 212 |
| Equine | Mr | 1 | Bovine V. Naked | Naked | 56 | 18 | 38 | 112 | 56 | 212 |
| Equine | Mr | 2 | Bovine V. Naked | Naked | 100 | 8 | 0 | 108 | 8 | 215 |
| Equine | Mr | 3 | Bovine V. Naked | Naked | 8 | 20 | 79 | 107 | 99 | 215 |
| Equine | Mr | 4 | Bovine V. Naked | Naked | 77 | 13 | 41 | 131 | 54 | 240 |
| Equine | Mr | 5 | Bovine V. Naked | Naked | 39 | 17 | 53 | 109 | 70 | 240 |
| Equine | Mr | 1 | Equine V. Bovine | Bovine | 52 | 21 | 23 | 96 | 44 | 241 |
| Equine | Mr | 2 | Equine V. Bovine | Bovine | 90 | 23 | 32 | 145 | 55 | 241 |
| Equine | Mr | 3 | Equine V. Bovine | Bovine | 60 | 51 | 35 | 146 | 86 | 285 |
| Equine | Mr | 4 | Equine V. Bovine | Bovine | 67 | 36 | 36 | 139 | 72 | 285 |
| Equine | Mr | 5 | Equine V. Bovine | Bovine | 57 | 34 | 26 | 117 | 60 | 210 |
| Equine | Mr | 1 | Equine V. Bovine | Equine | 61 | 6 | 26 | 93 | 32 | 210 |
| Equine | Mr | 2 | Equine V. Bovine | Equine | 64 | 24 | 16 | 104 | 40 | 202 |
| Equine | Mr | 3 | Equine V. Bovine | Equine | 58 | 1 | 39 | 98 | 40 | 202 |
| Equine | Mr | 4 | Equine V. Bovine | Equine | 62 | 3 | 35 | 100 | 38 | 234 |
| Equine | Mr | 5 | Equine V. Bovine | Equine | 108 | 6 | 20 | 134 | 26 | 234 |
| Equine | Mr | 1 | Equine V. Naked | Equine | 72 | 3 | 30 | 105 | 33 | 207 |
| Equine | Mr | 2 | Equine V. Naked | Equine | 57 | 3 | 42 | 102 | 45 | 207 |
| Equine | Mr | 3 | Equine V. Naked | Equine | 60 | 24 | 21 | 105 | 45 | 224 |
| Equine | Mr | 4 | Equine V. Naked | Equine | 68 | 48 | 3 | 119 | 51 | 224 |
| Equine | Mr | 5 | Equine V. Naked | Equine | 68 | 23 | 34 | 125 | 57 | 244 |
| Equine | Mr | 1 | Equine V. Naked | Naked | 90 | 6 | 23 | 119 | 29 | 244 |
| Equine | Mr | 2 | Equine V. Naked | Naked | 52 | 7 | 51 | 110 | 58 | 220 |
| Equine | Mr | 3 | Equine V. Naked | Naked | 66 | 4 | 40 | 110 | 44 | 220 |
| Equine | Mr | 4 | Equine V. Naked | Naked | 88 | 6 | 36 | 130 | 42 | 240 |
| Equine | Mr | 5 | Equine V. Naked | Naked | 64 | 6 | 40 | 110 | 46 | 240 |
| Equine | Mr | 1 | Naked V. Naked | Control | 35 | 29 | 59 | 123 | 88 | 237 |
| Equine | Mr | 1 | Naked V. Naked | Control | 38 | 19 | 57 | 114 | 76 | 237 |
| Equine | Mr | 2 | Naked V. Naked | Control | 66 | 12 | 28 | 106 | 40 | 215 |
| Equine | Mr | 2 | Naked V. Naked | Control | 10 | 6 | 93 | 109 | 99 | 215 |
| Equine | Mr | 3 | Naked V. Naked | Control | 25 | 24 | 63 | 112 | 87 | 218 |
| Equine | Mr | 3 | Naked V. Naked | Control | 25 | 20 | 61 | 106 | 81 | 218 |
| Equine | Mr | 4 | Naked V. Naked | Control | 96 | 11 | 48 | 155 | 59 | 295 |
| Equine | Mr | 4 | Naked V. Naked | Control | 96 | 2 | 42 | 140 | 44 | 295 |
| Equine | Mr | 5 | Naked V. Naked | Control | 8 | 28 | 70 | 106 | 98 | 249 |
| Equine | Mr | 5 | Naked V. Naked | Control | 85 | 12 | 46 | 143 | 58 | 249 |
| Naked | Mr | 1 | Bovine V. Naked | Bovine | 92 | 0 | 40 | 132 | 40 | 266 |
| Naked | Mr | 2 | Bovine V. Naked | Bovine | 88 | 19 | 27 | 134 | 46 | 266 |
| Naked | Mr | 3 | Bovine V. Naked | Bovine | 87 | 5 | 26 | 118 | 31 | 246 |
| Naked | Mr | 4 | Bovine V. Naked | Bovine | 77 | 23 | 28 | 128 | 51 | 246 |
| Naked | Mr | 5 | Bovine V. Naked | Bovine | 49 | 23 | 27 | 99 | 50 | 201 |
| Naked | Mr | 1 | Bovine V. Naked | Naked | 100 | 2 | 0 | 102 | 2 | 201 |
| Naked | Mr | 2 | Bovine V. Naked | Naked | 26 | 25 | 48 | 99 | 73 | 213 |
| Naked | Mr | 3 | Bovine V. Naked | Naked | 24 | 19 | 71 | 114 | 90 | 213 |
| Naked | Mr | 4 | Bovine V. Naked | Naked | 37 | 11 | 56 | 104 | 67 | 203 |
| Naked | Mr | 5 | Bovine V. Naked | Naked | 2 | 20 | 77 | 99 | 97 | 203 |
| Naked | Mr | 1 | Equine V. Bovine | Bovine | 77 | 9 | 48 | 134 | 57 | 268 |
| Naked | Mr | 2 | Equine V. Bovine | Bovine | 55 | 29 | 50 | 134 | 79 | 268 |
| Naked | Mr | 3 | Equine V. Bovine | Bovine | 75 | 17 | 24 | 116 | 41 | 232 |
| Naked | Mr | 4 | Equine V. Bovine | Bovine | 46 | 20 | 50 | 116 | 70 | 232 |
| Naked | Mr | 5 | Equine V. Bovine | Bovine | 17 | 33 | 43 | 93 | 76 | 200 |
| Naked | Mr | 1 | Equine V. Bovine | Equine | 76 | 0 | 31 | 107 | 31 | 200 |
| Naked | Mr | 2 | Equine V. Bovine | Equine | 71 | 12 | 32 | 115 | 44 | 224 |
| Naked | Mr | 3 | Equine V. Bovine | Equine | 62 | 31 | 16 | 109 | 47 | 224 |
| Naked | Mr | 4 | Equine V. Bovine | Equine | 79 | 8 | 26 | 113 | 34 | 234 |
| Naked | Mr | 5 | Equine V. Bovine | Equine | 77 | 22 | 22 | 121 | 44 | 234 |
| Naked | Mr | 1 | Equine V. Naked | Equine | 72 | 15 | 26 | 113 | 41 | 245 |
| Naked | Mr | 2 | Equine V. Naked | Equine | 76 | 20 | 36 | 132 | 56 | 245 |
| Naked | Mr | 3 | Equine V. Naked | Equine | 67 | 13 | 19 | 99 | 32 | 212 |
| Naked | Mr | 4 | Equine V. Naked | Equine | 81 | 9 | 23 | 113 | 32 | 212 |
| Naked | Mr | 5 | Equine V. Naked | Equine | 76 | 14 | 6 | 96 | 20 | 232 |
| Naked | Mr | 1 | Equine V. Naked | Naked | 80 | 6 | 50 | 136 | 56 | 232 |
| Naked | Mr | 2 | Equine V. Naked | Naked | 7 | 23 | 73 | 103 | 96 | 208 |
| Naked | Mr | 3 | Equine V. Naked | Naked | 2 | 22 | 81 | 105 | 103 | 208 |
| Naked | Mr | 4 | Equine V. Naked | Naked | 3 | 20 | 81 | 104 | 101 | 204 |
| Naked | Mr | 5 | Equine V. Naked | Naked | 7 | 20 | 73 | 100 | 93 | 204 |
| Naked | Mr | 1 | Naked V. Naked | Control | 36 | 6 | 90 | 132 | 96 | 262 |
| Naked | Mr | 1 | Naked V. Naked | Control | 90 | 8 | 32 | 130 | 38 | 262 |
| Naked | Mr | 2 | Naked V. Naked | Control | 3 | 17 | 85 | 105 | 102 | 221 |
| Naked | Mr | 2 | Naked V. Naked | Control | 40 | 23 | 53 | 116 | 76 | 221 |
| Naked | Mr | 3 | Naked V. Naked | Control | 39 | 13 | 66 | 118 | 79 | 227 |
| Naked | Mr | 3 | Naked V. Naked | Control | 3 | 35 | 71 | 109 | 106 | 227 |
| Naked | Mr | 4 | Naked V. Naked | Control | 7 | 18 | 84 | 109 | 102 | 219 |
| Naked | Mr | 4 | Naked V. Naked | Control | 28 | 18 | 64 | 110 | 82 | 219 |
| Naked | Mr | 5 | Naked V. Naked | Control | 0 | 27 | 88 | 115 | 115 | 215 |
| Naked | Mr | 5 | Naked V. Naked | Control | 5 | 16 | 79 | 100 | 95 | 215 |
| Bovine | Sc | 1 | Bovine V. Naked | Bovine | 2 | 23 | 21 | 46 | 53 | 186 |
| Bovine | Sc | 2 | Bovine V. Naked | Bovine | 14 | 86 | 40 | 140 | 100 | 186 |
| Bovine | Sc | 3 | Bovine V. Naked | Bovine | 18 | 21 | 25 | 64 | 49 | 207 |
| Bovine | Sc | 4 | Bovine V. Naked | Bovine | 5 | 76 | 62 | 143 | 113 | 207 |
| Bovine | Sc | 5 | Bovine V. Naked | Bovine | 8 | 89 | 83 | 180 | 124 | 285 |
| Bovine | Sc | 1 | Bovine V. Naked | Naked | 78 | 18 | 9 | 105 | 27 | 285 |
| Bovine | Sc | 2 | Bovine V. Naked | Naked | 0 | 85 | 12 | 97 | 97 | 192 |
| Bovine | Sc | 3 | Bovine V. Naked | Naked | 59 | 12 | 24 | 95 | 36 | 192 |
| Bovine | Sc | 4 | Bovine V. Naked | Naked | 5 | 73 | 30 | 108 | 103 | 214 |
| Bovine | Sc | 5 | Bovine V. Naked | Naked | 1 | 91 | 14 | 106 | 105 | 214 |
| Bovine | Sc | 1 | Equine V. Bovine | Bovine | 12 | 29 | 28 | 69 | 57 | 173 |
| Bovine | Sc | 2 | Equine V. Bovine | Bovine | 12 | 55 | 37 | 104 | 92 | 173 |
| Bovine | Sc | 3 | Equine V. Bovine | Bovine | 27 | 19 | 35 | 81 | 54 | 202 |
| Bovine | Sc | 4 | Equine V. Bovine | Bovine | 18 | 67 | 36 | 121 | 103 | 202 |
| Bovine | Sc | 5 | Equine V. Bovine | Bovine | 11 | 65 | 31 | 107 | 96 | 151 |
| Bovine | Sc | 1 | Equine V. Bovine | Equine | 1 | 5 | 38 | 44 | 43 | 151 |
| Bovine | Sc | 2 | Equine V. Bovine | Equine | 0 | 6 | 61 | 67 | 67 | 157 |
| Bovine | Sc | 3 | Equine V. Bovine | Equine | 3 | 9 | 78 | 90 | 87 | 157 |
| Bovine | Sc | 4 | Equine V. Bovine | Equine | 5 | 13 | 102 | 120 | 115 | 221 |
| Bovine | Sc | 5 | Equine V. Bovine | Equine | 0 | 32 | 69 | 101 | 101 | 221 |
| Bovine | Sc | 1 | Equine V. Naked | Equine | 3 | 34 | 30 | 67 | 64 | 132 |
| Bovine | Sc | 2 | Equine V. Naked | Equine | 2 | 6 | 57 | 65 | 63 | 132 |
| Bovine | Sc | 3 | Equine V. Naked | Equine | 2 | 13 | 65 | 80 | 78 | 179 |
| Bovine | Sc | 4 | Equine V. Naked | Equine | 2 | 27 | 70 | 99 | 97 | 179 |
| Bovine | Sc | 5 | Equine V. Naked | Equine | 0 | 42 | 58 | 100 | 100 | 200 |
| Bovine | Sc | 1 | Equine V. Naked | Naked | 40 | 33 | 27 | 100 | 60 | 200 |
| Bovine | Sc | 2 | Equine V. Naked | Naked | 0 | 83 | 20 | 103 | 103 | 206 |
| Bovine | Sc | 3 | Equine V. Naked | Naked | 46 | 25 | 32 | 103 | 57 | 206 |
| Bovine | Sc | 4 | Equine V. Naked | Naked | 5 | 80 | 12 | 97 | 92 | 209 |
| Bovine | Sc | 5 | Equine V. Naked | Naked | 6 | 104 | 2 | 112 | 106 | 209 |
| Bovine | Sc | 1 | Naked V. Naked | Control | 61 | 18 | 15 | 94 | 33 | 193 |
| Bovine | Sc | 1 | Naked V. Naked | Control | 77 | 14 | 8 | 99 | 22 | 193 |
| Bovine | Sc | 2 | Naked V. Naked | Control | 3 | 86 | 10 | 99 | 96 | 191 |
| Bovine | Sc | 2 | Naked V. Naked | Control | 1 | 68 | 23 | 92 | 91 | 191 |
| Bovine | Sc | 3 | Naked V. Naked | Control | 62 | 20 | 18 | 100 | 38 | 203 |
| Bovine | Sc | 3 | Naked V. Naked | Control | 32 | 32 | 39 | 103 | 71 | 203 |
| Bovine | Sc | 4 | Naked V. Naked | Control | 6 | 90 | 7 | 103 | 97 | 209 |
| Bovine | Sc | 4 | Naked V. Naked | Control | 5 | 100 | 1 | 106 | 101 | 209 |
| Bovine | Sc | 5 | Naked V. Naked | Control | 5 | 81 | 42 | 128 | 123 | 235 |
| Bovine | Sc | 5 | Naked V. Naked | Control | 2 | 82 | 23 | 107 | 105 | 235 |
| Equine | Sc | 1 | Bovine V. Naked | Bovine | 3 | 14 | 37 | 54 | 51 | 108 |
| Equine | Sc | 2 | Bovine V. Naked | Bovine | 31 | 14 | 9 | 54 | 23 | 108 |
| Equine | Sc | 3 | Bovine V. Naked | Bovine | 7 | 48 | 60 | 115 | 108 | 227 |
| Equine | Sc | 4 | Bovine V. Naked | Bovine | 27 | 40 | 45 | 112 | 85 | 227 |
| Equine | Sc | 5 | Bovine V. Naked | Bovine | 8 | 71 | 40 | 119 | 111 | 209 |
| Equine | Sc | 1 | Bovine V. Naked | Naked | 48 | 36 | 6 | 90 | 42 | 209 |
| Equine | Sc | 2 | Bovine V. Naked | Naked | 67 | 34 | 9 | 110 | 43 | 210 |
| Equine | Sc | 3 | Bovine V. Naked | Naked | 46 | 26 | 28 | 100 | 54 | 210 |
| Equine | Sc | 4 | Bovine V. Naked | Naked | 52 | 33 | 20 | 105 | 53 | 240 |
| Equine | Sc | 5 | Bovine V. Naked | Naked | 82 | 23 | 30 | 135 | 53 | 240 |
| Equine | Sc | 1 | Equine V. Bovine | Bovine | 9 | 20 | 20 | 49 | 40 | 95 |
| Equine | Sc | 2 | Equine V. Bovine | Bovine | 25 | 15 | 6 | 46 | 21 | 95 |
| Equine | Sc | 3 | Equine V. Bovine | Bovine | 25 | 54 | 28 | 107 | 82 | 212 |
| Equine | Sc | 4 | Equine V. Bovine | Bovine | 21 | 42 | 42 | 105 | 84 | 212 |
| Equine | Sc | 5 | Equine V. Bovine | Bovine | 44 | 18 | 68 | 130 | 86 | 214 |
| Equine | Sc | 1 | Equine V. Bovine | Equine | 25 | 11 | 48 | 84 | 59 | 214 |
| Equine | Sc | 2 | Equine V. Bovine | Equine | 6 | 5 | 44 | 55 | 49 | 143 |
| Equine | Sc | 3 | Equine V. Bovine | Equine | 10 | 10 | 68 | 88 | 78 | 143 |
| Equine | Sc | 4 | Equine V. Bovine | Equine | 3 | 16 | 59 | 78 | 75 | 229 |
| Equine | Sc | 5 | Equine V. Bovine | Equine | 8 | 46 | 97 | 151 | 143 | 229 |
| Equine | Sc | 1 | Equine V. Naked | Equine | 6 | 20 | 48 | 74 | 68 | 120 |
| Equine | Sc | 2 | Equine V. Naked | Equine | 9 | 12 | 25 | 46 | 37 | 120 |
| Equine | Sc | 3 | Equine V. Naked | Equine | 4 | 16 | 71 | 91 | 87 | 174 |
| Equine | Sc | 4 | Equine V. Naked | Equine | 4 | 20 | 59 | 83 | 79 | 174 |
| Equine | Sc | 5 | Equine V. Naked | Equine | 3 | 7 | 79 | 89 | 86 | 235 |
| Equine | Sc | 1 | Equine V. Naked | Naked | 57 | 20 | 69 | 146 | 89 | 235 |
| Equine | Sc | 2 | Equine V. Naked | Naked | 6 | 29 | 36 | 71 | 65 | 171 |
| Equine | Sc | 3 | Equine V. Naked | Naked | 34 | 24 | 42 | 100 | 66 | 171 |
| Equine | Sc | 4 | Equine V. Naked | Naked | 71 | 11 | 19 | 101 | 30 | 213 |
| Equine | Sc | 5 | Equine V. Naked | Naked | 54 | 58 | 0 | 112 | 58 | 213 |
| Equine | Sc | 1 | Naked V. Naked | Control | 34 | 40 | 22 | 96 | 62 | 188 |
| Equine | Sc | 1 | Naked V. Naked | Control | 25 | 48 | 19 | 92 | 67 | 188 |
| Equine | Sc | 2 | Naked V. Naked | Control | 70 | 22 | 13 | 105 | 35 | 210 |
| Equine | Sc | 2 | Naked V. Naked | Control | 31 | 36 | 38 | 105 | 74 | 210 |
| Equine | Sc | 3 | Naked V. Naked | Control | 12 | 51 | 41 | 104 | 92 | 214 |
| Equine | Sc | 3 | Naked V. Naked | Control | 77 | 24 | 9 | 110 | 33 | 214 |
| Equine | Sc | 4 | Naked V. Naked | Control | 17 | 53 | 32 | 102 | 85 | 210 |
| Equine | Sc | 4 | Naked V. Naked | Control | 12 | 62 | 34 | 108 | 96 | 210 |
| Equine | Sc | 5 | Naked V. Naked | Control | 77 | 9 | 19 | 105 | 28 | 214 |
| Equine | Sc | 5 | Naked V. Naked | Control | 51 | 22 | 36 | 109 | 58 | 214 |
| Naked | Sc | 1 | Bovine V. Naked | Bovine | 22 | 33 | 20 | 75 | 53 | 167 |
| Naked | Sc | 2 | Bovine V. Naked | Bovine | 6 | 81 | 5 | 92 | 86 | 167 |
| Naked | Sc | 3 | Bovine V. Naked | Bovine | 18 | 56 | 33 | 107 | 89 | 226 |
| Naked | Sc | 4 | Bovine V. Naked | Bovine | 4 | 61 | 54 | 119 | 115 | 226 |
| Naked | Sc | 5 | Bovine V. Naked | Bovine | 7 | 22 | 80 | 109 | 102 | 210 |
| Naked | Sc | 1 | Bovine V. Naked | Naked | 22 | 73 | 6 | 101 | 79 | 210 |
| Naked | Sc | 2 | Bovine V. Naked | Naked | 11 | 90 | 1 | 102 | 91 | 192 |
| Naked | Sc | 3 | Bovine V. Naked | Naked | 2 | 78 | 10 | 90 | 88 | 192 |
| Naked | Sc | 4 | Bovine V. Naked | Naked | 4 | 63 | 36 | 103 | 99 | 206 |
| Naked | Sc | 5 | Bovine V. Naked | Naked | 99 | 4 | 0 | 103 | 4 | 206 |
| Naked | Sc | 1 | Equine V. Bovine | Bovine | 32 | 38 | 21 | 91 | 59 | 193 |
| Naked | Sc | 2 | Equine V. Bovine | Bovine | 5 | 73 | 24 | 102 | 97 | 193 |
| Naked | Sc | 3 | Equine V. Bovine | Bovine | 20 | 40 | 16 | 76 | 56 | 203 |
| Naked | Sc | 4 | Equine V. Bovine | Bovine | 43 | 46 | 38 | 127 | 84 | 203 |
| Naked | Sc | 5 | Equine V. Bovine | Bovine | 50 | 27 | 41 | 118 | 68 | 193 |
| Naked | Sc | 1 | Equine V. Bovine | Equine | 11 | 22 | 42 | 75 | 64 | 193 |
| Naked | Sc | 2 | Equine V. Bovine | Equine | 7 | 41 | 78 | 126 | 119 | 232 |
| Naked | Sc | 3 | Equine V. Bovine | Equine | 17 | 41 | 48 | 106 | 89 | 232 |
| Naked | Sc | 4 | Equine V. Bovine | Equine | 4 | 16 | 75 | 95 | 91 | 214 |
| Naked | Sc | 5 | Equine V. Bovine | Equine | 44 | 36 | 39 | 119 | 75 | 214 |
| Naked | Sc | 1 | Equine V. Naked | Equine | 36 | 44 | 38 | 118 | 82 | 257 |
| Naked | Sc | 2 | Equine V. Naked | Equine | 8 | 39 | 92 | 139 | 131 | 257 |
| Naked | Sc | 3 | Equine V. Naked | Equine | 0 | 12 | 48 | 60 | 60 | 163 |
| Naked | Sc | 4 | Equine V. Naked | Equine | 4 | 9 | 90 | 103 | 99 | 163 |
| Naked | Sc | 5 | Equine V. Naked | Equine | 13 | 11 | 79 | 103 | 90 | 216 |
| Naked | Sc | 1 | Equine V. Naked | Naked | 23 | 89 | 1 | 113 | 90 | 216 |
| Naked | Sc | 2 | Equine V. Naked | Naked | 5 | 89 | 1 | 95 | 90 | 189 |
| Naked | Sc | 3 | Equine V. Naked | Naked | 6 | 83 | 5 | 94 | 88 | 189 |
| Naked | Sc | 4 | Equine V. Naked | Naked | 15 | 71 | 9 | 95 | 80 | 209 |
| Naked | Sc | 5 | Equine V. Naked | Naked | 107 | 7 | 0 | 114 | 7 | 209 |
| Naked | Sc | 1 | Naked V. Naked | Control | 14 | 67 | 21 | 102 | 88 | 197 |
| Naked | Sc | 1 | Naked V. Naked | Control | 9 | 68 | 18 | 95 | 86 | 197 |
| Naked | Sc | 2 | Naked V. Naked | Control | 6 | 88 | 5 | 99 | 93 | 193 |
| Naked | Sc | 2 | Naked V. Naked | Control | 2 | 68 | 24 | 94 | 92 | 193 |
| Naked | Sc | 3 | Naked V. Naked | Control | 5 | 61 | 22 | 88 | 83 | 202 |
| Naked | Sc | 3 | Naked V. Naked | Control | 4 | 82 | 28 | 114 | 110 | 202 |
| Naked | Sc | 4 | Naked V. Naked | Control | 8 | 71 | 25 | 104 | 96 | 210 |
| Naked | Sc | 4 | Naked V. Naked | Control | 7 | 83 | 16 | 106 | 99 | 210 |
| Naked | Sc | 5 | Naked V. Naked | Control | 82 | 17 | 15 | 114 | 32 | 222 |
| Naked | Sc | 5 | Naked V. Naked | Control | 48 | 19 | 41 | 108 | 60 | 222 |
